# The Ion Channel and GPCR Toolkit of Brain Capillary Pericytes

**DOI:** 10.3389/fncel.2020.601324

**Published:** 2020-12-18

**Authors:** Ashwini Hariharan, Nick Weir, Colin Robertson, Liqun He, Christer Betsholtz, Thomas A. Longden

**Affiliations:** ^1^Department of Physiology, School of Medicine, University of Maryland, Baltimore, MD, United States; ^2^Rudbeck Laboratory, Department of Immunology, Genetics and Pathology, Uppsala University, Uppsala, Sweden; ^3^Department of Medicine Huddinge (MedH), Karolinska Institutet & Integrated Cardio Metabolic Centre, Huddinge, Sweden

**Keywords:** pericytes, ion channels, GPCRs (G protein coupled receptors), neurovascular coupling (NVC), cerebral blood flow (CBF), K_ATP_ channels, brain metabolism

## Abstract

Brain pericytes reside on the abluminal surface of capillaries, and their processes cover ~90% of the length of the capillary bed. These cells were first described almost 150 years ago (Eberth, 1871; Rouget, 1873) and have been the subject of intense experimental scrutiny in recent years, but their physiological roles remain uncertain and little is known of the complement of signaling elements that they employ to carry out their functions. In this review, we synthesize functional data with single-cell RNAseq screens to explore the ion channel and G protein-coupled receptor (GPCR) toolkit of mesh and thin-strand pericytes of the brain, with the aim of providing a framework for deeper explorations of the molecular mechanisms that govern pericyte physiology. We argue that their complement of channels and receptors ideally positions capillary pericytes to play a central role in adapting blood flow to meet the challenge of satisfying neuronal energy requirements from deep within the capillary bed, by enabling dynamic regulation of their membrane potential to influence the electrical output of the cell. In particular, we outline how genetic and functional evidence suggest an important role for G_s_-coupled GPCRs and ATP-sensitive potassium (K_ATP_) channels in this context. We put forth a predictive model for long-range hyperpolarizing electrical signaling from pericytes to upstream arterioles, and detail the TRP and Ca^2+^ channels and G_q_, G_i/o_, and G_12/13_ signaling processes that counterbalance this. We underscore critical questions that need to be addressed to further advance our understanding of the signaling topology of capillary pericytes, and how this contributes to their physiological roles and their dysfunction in disease.

## Introduction

A combination of autonomic signaling (Cipolla et al., 2004; Hamel, 2006) and intrinsic pressure sensing and metabolic autoregulatory mechanisms (Bayliss, 1902; Paulson et al., 1990) drives continual adjustments in global and local blood flow in the brain. Importantly, as the brain lacks substantial energy stores it must be able to rapidly adapt local blood flow to fluctuating neuronal metabolic needs to provide adequate oxygen and glucose delivery. This is achieved through the on-demand process of functional hyperemia (FH), where increases in neural activity—which can span orders of magnitude in milliseconds—are met with an increase in local blood flow within seconds. This call-and-response phenomenon is underlain by a complex range of stratified mechanisms, collectively termed neurovascular coupling (NVC), which have inbuilt redundancy to ensure the fidelity of the blood flow response.

Significant inroads toward a full understanding of these NVC mechanisms have been made in recent years (Iadecola, 2017), and in particular ion channel and GPCR signaling networks within and between the cells of the neurovascular unit [NVU; neurons, astrocytes, smooth muscle cells (SMCs), endothelial cells (ECs), and pericytes] are emerging as major contributors (Longden et al., 2016). However, capillary pericytes represent a relative blind spot in our knowledge, and our understanding of their involvement in brain blood flow control is less well-developed than that for other cells of the NVU. Accordingly, the purpose of this review is to survey the signaling toolkit that mesh and thin-strand pericytes may employ to contribute to the control of blood flow throughout the brain. To this end, we leverage data from recent brain single-cell RNAseq (scRNAseq) screens (He et al., 2018; Vanlandewijck et al., 2018; Zeisel et al., 2018) to profile the expression of ion channels (Table 1) and GPCRs (Table 2) in brain capillary pericytes which, when synthesized with functional results, may aid in delineating their physiological roles.

**Table 1:** Ion channels expressed by CNS capillary pericytes.

**Channel protein**	**Gene**	mRNA average counts/cell^*^	**Ion selectivity**	**Endogenous activators and key modulators**	**Key properties**	**Key references**
K_ir_6.1	*Kcnj8*	1670.21	K^+^	ATP:ADP, UDP, G_q_/G_s_ signaling	Weakly rectifying; Forms K_ATP_ channel with SUR2 to mediate metabolism-electrical coupling	Ishizaki et al., 2009
K_ir_2.2	*Kcnj12*	31.01	K^+^	K^+^, hyperpolarization	Strongly rectifying; Propagation of hyperpolarizing signals	Matsushita and Puro, 2006; Longden and Nelson, 2015; Longden et al., 2017
K_v_1.2	*Kcna2*	1.25	K^+^	Depolarization	Negative feedback regulation of V_m_; K_v_ currents have been reported in peripheral pericytes	Nelson and Quayle, 1995; von Beckerath et al., 2000; Quignard et al., 2003
K_v_2.1	*Kcnb1*	4.11	K^+^			
K_v_6.1	*Kcng1*	8.75	K^+^			
K_v_7.4	*Kcnq4*	7.7	K^+^			
K_v_7.5	*Kcnq5*	1.48	K^+^			
K_v_9.1	*Kcns1*	1.66	K^+^			
K_v_9.3	*Kcns3*	3.21	K^+^			
K_2P_3.1	*Kcnk3*	9.8	K^+^	pH	Activation in response to moderate rise in pH	Duprat et al., 1997
K_Na_1.2	*Kcnt2*	5.87	K^+^	Intracellular Na^+^, Cl^−^	Maintaining resting V_m_; Sensitive to cell volume changes; Inactivated by ADP and ATP	Bhattacharjee et al., 2003; Tejada et al., 2014
K_Ca_2.3	*Kcnn3*	1.81	K^+^	Intracellular Ca^2+^	Hyperpolarization in response to Ca^2+^ elevation;	Taylor et al., 2003; Adelman et al., 2012
TRPC1	*Trpc1*	16.04	Na^+^, K^+^, Ca^2+^	n.d^a^	Store-operated Ca^2+^ entry in association with STIM1 and Orai1	Huang et al., 2006; Cheng et al., 2008
TRPC3	*Trpc3*	266.99	***p*****Ca**^**2+**^**/*****p*****Na**^**+**^**: 1.6** Na^+^, K^+^, Ca^2+^	G_q_ signaling, DAG	Facilitates Ca^2+^ entry; Depolarizes V_m_	Xi et al., 2009; Kochukov et al., 2014
TRPC4	*Trpc4*	67.83	***p*****Ca**^**2+**^**/*****p*****Na**^**+**^**: 1.1-7.7** Na^+^, K^+^, Ca^2+^	G_i/o_/G_q_ signaling	Activated by G_i/o_-GPCR signaling	Albert, 2011; Jeon et al., 2012
TRPC6	*Trpc6*	5.94	***p*****Ca**^**2+**^**/*****p*****Na**^**+**^**: 5** Na^+^, K^+^, Ca^2+^	G_q_ signaling, arachidonic acid, lysophosphatidylcholine, 20-HETE	Mechanosensation; Ca^2+^ influx through TRPC6 can sensitize IP_3_R to cause Ca^2+^ release	Gonzales et al., 2014
TRPM3	*Trpm3*	1.04	***p*****Ca**^**2+**^**/*****p*****Na**^**+**^**: 1.6** Na^+^, K^+^, Ca^2+^, Mg^2+^^**^	Sphingosine, sphinganine, NN-dimethyl-D-erythrosphingosine, pregnenolone sulfate	Steroid signaling; Lipid signaling; Mechanosensation	Grimm et al., 2005; Wagner et al., 2008
TRPM4	*Trpm4*	21.32	Na^+^, K^+^	PIP_2_, intracellular Ca^2+^	Permeable to monovalent cations; Depolarizes V_m_ in response to Ca^2+^ elevations	Gonzales et al., 2014
TRPM7	*Trpm7*	104.35	***p*****Ca**^**2+**^**/*****p*****Na**^**+**^**: 0.34** Na^+^, K^+^, Ca^2+^, Mg^2+^	PIP_2_	Mg^2+^ homeostasis; Can modulate store-operated Ca^2+^ entry; pH sensitive;	Schlingmann et al., 2007; Souza Bomfim et al., 2020
TRPML1	*Mcoln1*	39.53	Na^+^, K^+^, Ca^2+^, Mg^2+^	Phosphatidyl (3,5) inositol bisphosphate	Lysosomal ion homeostasis	Venkatachalam et al., 2015
TRPP1	*Pkd2*	117.24	Na^+^, K^+^, Ca^2+^	Intracellular Ca^2+^	Large Ca^2+^ conductance; Mechanosensation in association with PKD1	Sharif-Naeini et al., 2009; Narayanan et al., 2013
TRPP3	*Pkd2l2*	1.3	***p*****Ca**^**2+**^**/*****p*****Na**^**+**^**: 4-4.3** Na^+^, K^+^, Ca^2+^, Mg^2+^	n.d	pH sensitive	Inada et al., 2008
TRPV2	*Trpv2*	98.6	***p*****Ca**^**2+**^**/*****p*****Na**^**+**^**: 0.9-2.9** Na^+^, K^+^, Ca^2+^, Mg^2+^	n.d	Mechanosensitive - detects cell swelling/stretch	Perálvarez-Marín et al., 2013
IP_3_R1	*Itpr1*	209.62	Ca^2+^	IP_3_, cytosolic Ca^2+^	Mediate Ca^2+^ release from endoplasmic reticulum upon binding of IP_3_; Participate in many intracellular Ca^2+^ signaling processes	Foskett et al., 2007; Berridge, 2016
IP_3_R2	*Itpr2*	250.43	Ca^2+^			
IP_3_R3	*Itpr3*	1.73	Ca^2+^			
Ca_v_1.2	*Cacna1c*	99.46	Ca^2+^	Depolarization	Ca^2+^ entry in response to depolarization or at resting; V_m_; L-type Ca^2+^ currents recorded in retinal pericytes	Sakagami et al., 1999; Perez-Reyes, 2003
Ca_v_1.3	*Cacna1d*	2.48				
Ca_v_2.1	*Cacna1a*	1.05				
Ca_v_3.1	*Cacna1g*	1.73				
Ca_v_3.2	*Cacna1h*	42.59				
Orai1	*Orai1*	22.88	Ca^2+^	ER Ca^2+^ depletion	Store operated Ca^2+^ entry channels; Associate with STIM1 to permit Ca^2+^ entry upon store depletion	Prakriya and Lewis, 2015
Orai3	*Orai3*	99.94				
CaCC (TMEM16A)	*Ano1*	329.91	Cl^−^	Intracellular Ca^2+^	Membrane depolarization in response to increased Ca^2+^; CaCC currents reported in retinal and peripheral pericytes	Sakagami et al., 1999; Hashitani et al., 2018
ClC-2	*Clcn2*	19.95	Cl^−^	Hyperpolarization, arachidonic acid	Repolarization of V_m_; Sensitive to intracellular ATP and ADP	Nilius and Droogmans, 2003; Stölting et al., 2013; Bi et al., 2014
ASIC2	*Asic2*	4.52	***p*****Na**^**+**^**/*****p*****Ca**^**2+**^**: 20** ***p*****Na**^**+**^**/*****p*****K**^**+**^**: 10** Na^+^, K^+^, Ca^2+^	Extracellular H^+^	Activated by extracellular acidification	Gannon et al., 2008; Sherwood et al., 2012
Na_v_1.2	*Scn2a*	3.02	Na^+^	Depolarization	Na^+^ influx in response to membrane depolarization; Na_v_1.3 is expressed in peripheral pericytes	Yu and Catterall, 2003; Lee-Kwon et al., 2007
Na_v_1.3	*Scn3a*	1.53	Na^+^			
P2X1	*P2rx1*	10.53	Na^+^, K^+^, Ca^2+^	ATP	Local ATP sensors	Khakh et al., 2001
P2X4	*P2rx4*	23.6				
Piezo1	*Piezo1*	2.09	Na^+^, K^+^, Ca^2+^, Mg^2+^	Mechanically activated	Senses and couples shear stress with cation entry	Coste et al., 2010; Li et al., 2014
TPC1	*Tpcn1*	36.44	Na^+^, K^+^, Ca^2+^	Phosphatidyl (3,5) inositol bisphosphate	Located on endosomal/lysosomal membranes; NAADP-induced Ca^2+^ release	Calcraft et al., 2009; Pitt et al., 2016
TPC2	*Tpcn2*	6.01				

*Naming conventions used throughout conform to those outlined in the IUPHAR/BPS Guide to Pharmacology (Armstrong et al., 2020). Permeability ratios are noted in bold where appropriate. Abbreviations not used elsewhere: NAADP, nicotinic acid adenine dinucleotide phosphate*.

a *n.d., no data*.

* *Data from He et al. (2018) and Vanlandewijck et al. (2018), expressed as average counts per cell annotated as a brain pericyte. Cells were isolated from adult mice of either sex aged 10–19 weeks*.

** *Permeability for short-pore sequence isoform TRPM3α2*.

**Table 2:** G protein coupled receptors expressed by CNS capillary pericytes.

**Receptor**	**Gene**	**mRNA** **average** counts/cell^*^	Principal G-protein^**^	**GPCR sub-class**	**Endogenous agonists**	**Signal transduction effects; roles**	**Key references**
**Adenosine receptors**							
A_1_ receptor	*Adora1*	1.96	G_i/o_	A	Adenosine	↓ cAMP; Arterial vasoconstriction	Borea et al., 2018
A_2A_ receptor	*Adora2a*	85.45	G_s_			↑ cAMP; Arterial vasorelaxation	
A_2B_ receptor	*Adora2b*	6.52	G_s_			↑ cAMP; Arterial vasorelaxation	
**Adrenoceptors**							
α_1A_-adrenoreceptor	*Adra1a*	1.44	G_q_	A	Epinephrine > norepinephrine	↑ IP_3_/DAG; Arterial vasoconstriction	Hieble and Ruffolo, 1997; Guimarães and Moura, 2001; Muszkat et al., 2005; Silva and Zanesco, 2010; de Oliveira et al., 2019
α_1B_-adrenoreceptor	*Adra1b*	1.29	G_q_				
α_2A_-adrenoreceptor	*Adra2a*	1.73	G_i/o_			↓ cAMP; Arterial vasoconstriction	
α_2B_-adrenoreceptor	*Adra2b*	2.03	G_i/o_				
β2-adrenoreceptor	*Adrb2*	1.65	G_s_			↑ cAMP; Vasodilation	
Calcitonin receptor-like receptor	*Calcrl*	37.46	G_s_	B	CGRP > Adrenomedullin	Non-functional alone, requires a RAMP. Likely colocalizes with RAMP2 to form AM_1_ receptors in pericytes	Poyner et al., 2002
Chemerin receptor 1	*Cmklr1*	3.49	G_i/o_	A	Resolvin E1 > Chemerin	↓ cAMP; Vasoconstrictor with a role in inflammation	De Henau et al., 2016; Kennedy et al., 2016
**Chemokine receptors**							
CCR9	*Ccr9*	25.5	G_i/o_	A	CCL25	↑ Ca^2+^; Activation of adaptive immune response; Leukocyte recruitment	Watts et al., 2013; Mazzotti et al., 2017
CXCR4	*Cxcr4*	1.44	G_i/o_		CXCL12		
CCRL2	*Ccrl2*	85.53	n.d^a^		CCL19	Anchors and presents chemerin to Cmklr1-expressing cells	
**Endothelin receptors**							
ET_A_ receptor	*Ednra*	236.01	G_q_	A	Endothelin-1 > endothelin-2 > endothelin-3	Vasoconstriction in SMCs; Extracellular matrix production and inflammation	Patel et al., 2014; Maguire and Davenport, 2015; Urtatiz and Van Raamsdonk, 2016
ET_B_ receptor	*Ednrb*	20.99	G_s_, G_i/o_, G_q_			↑ IP_3_/DAG/PLA_2_/PLD; Vasodilation in ECs, vasoconstriction in SMCs	
FFA2 receptor	*Ffar2*	6.47	G_q_	A	Free fatty acids	↑ IP_3_/DAG; Roles in metabolism and inflammation	Li et al., 2018
GIP receptor	*Gipr*	8.48	G_s_	B	Gastric inhibitory polypeptide	↑ cAMP; Increases blood flow in adipose microvessels	Asmar et al., 2019
GPER	*Gper1*	716.19	G_i/o_	A	17β-estradiol	Diverse genomic and non-genomic roles; Vasodilation, likely via secondary G_s_ coupling	Prossnitz and Arterburn, 2015; Evans et al., 2016
Kisspeptin receptor	*Kiss1r*	1.93	G_q_	A	Kisspeptin-10,−13,−14,−54,−52	↑ IP_3_/DAG; Vasoconstrictor, inhibits angiogenesis	Sawyer et al., 2011; Cvetković et al., 2013
**Leukotriene receptors**							
CysLT_1_	*Cysltr1*	9	G_q_	A	LTD_4_ > LTC_4_ > LTE_4_ LTC_4_ > LTD_4_ > LTE_4_	↑ IP_3_/DAG; Vascular permeability, SMC contraction, immune cell activation	Zhang et al., 2006; Woszczek et al., 2007; Thiriet, 2013
CysLT_2_	*Cysltr2*	35.81	G_q_				
**Lysophospholipid receptors**							
LPA_1_	*Lpar1*	8.29	G_i/o_, G_q_, G_12/13_	A	LPA	↓ cAMP; ↑IP_3_/DAG and PLA_2_; Vasoconstrictor	Means and Brown, 2009; Cheng et al., 2012; Aoki et al., 2016; Pébay and Wong, 2017; Masago et al., 2018
LPA_6_	*Lpar6*	19.76	G_12/13_	A	LPA	↑ cAMP; ↑ IP_3_/DAG; BBB permeability	
S1P_1_	*S1pr1*	5.88	G_i/o_	A	S1P > sphingosylphosphoryl-choline > LPA	↓ cAMP; ↑ IP3/DAG and PLD; Leukocyte recruitment, ↓ vascular permeability	
S1P_2_	*S1pr2*	20.32	G_i/o_, G_q_, G_12/13_	A	S1P > sphingosylphosphoryl-choline	↑ cAMP; ↑ IP_3_/DAG; ↓ chemotaxis, ↑ vascular permeability	
S1P_3_	*S1pr3*	936.18	G_i/o_, G_q_, G_12/13_	A	S1P > sphingosylphosphoryl-choline	↓ cAMP; ↑ IP_3_/DAG; Vasoconstriction via SMCs, vasorelaxation via ECs; Angiogenesis	
**Metabotropic glutamate receptors**							
mGlu_3_ receptor	*Grm3*	206.24	G_i/o_	C	Glutamate > NAAG	↓ cAMP; Inhibits glial non-vesicular glutamate release and neuronal synaptic plasticity	Wroblewska et al., 1998; Harrison et al., 2008; Palazzo et al., 2016; Yudin and Rohacs, 2018
mGlu_7_ receptor	*Grm7*	94.26	G_i/o_	C	Glutamate > L-serine-O-phosphate	↓ cAMP; Low glutamate affinity, auto-inhibition of glutamate release	
NOP receptor	*Oprl1*	12.02	G_i/o_	A	Nociceptin/orphanin FQ	↓ cAMP; Bradycardia, hypotension upon systemic administration of agonist	Kapusta et al., 2002
PAC_1_ receptor	*Adcyap1r1*	35.51	G_s_, G_q_	B	PACAP-27 = PACAP-38 > VIP, PHI, PHM, PHV	↑ cAMP; Potent vasodilator	May et al., 2010; Koide et al., 2014
PAR1	*F2r*	141.17	G_i/o_, G_q_, G_12/13_	A	Thrombin activated protein C, MMP1, MMP13	Haematopoietic development, vascular development, peripheral vasodilation, hypotension, bradycardia	Cheung et al., 1998; Yue et al., 2012
PTH1 receptor	*Pth1r*	226.03	G_s_	B	PTH = PTHrP-1, TIP39	↑ cAMP; Systemic mineral homeostasis	Mahon, 2012
**Prostanoid receptors**							
DP_2_ receptor	*Ptgdr2*	2	G_i/o_	A	PGD_2_ > PGF_2α_ > PGE_2_ > PGI_2_, thromboxane A_2_ PGD_3_, PGJ_2_	↓ cAMP; Vasodilation, role in angiogenesis	Praticò and Dogné, 2005; Kaczynski et al., 2016; Longden et al., 2019; Upchurch and Leitinger, 2019; Ozen et al., 2020
EP_1_ receptor	*Ptger1*	10.87	G_q_	A	PGE_2_ > PGE_1_ > PGF_2α_ > PGI_2_ > PGD_2_ > thromboxane A_2_	↑ IP_3_/DAG; Role in NVC	
EP_3_ receptor	*Ptger3*	5.74	G_i/o_	A	PGE_2_ > PGE_1_ > PGF_2α_ > PGI_2_ > PGD_2_ > thromboxane A_2_	↓ cAMP	
FP receptor	*Ptgfr*	13.26	G_q_	A	PGF_2α_ > PGD_2_ > PGE_2_, PGI_2_ > thromboxane A_2_	↑ IP_3_/DAG; Angiogenesis, matrix remodeling	
IP receptor	*Ptgir*	2.7	G_s_	A	PGI_2_ > PGE_1_ > PGD_2_, PGF_2α_ > thromboxane A_2_ PGE_2_	↑ cAMP; Released from ECs, drives vasodilation, angiogenesis	
TP receptor	*Tbxa2r*	282.65	G_q_	A	Thromboxane A_2_ = PGH_2_ > PGD_2_, PGE_2_, PGF_2α_, PGI_2_,	↑ IP_3_/DAG; vasoconstriction	
**Purinergic receptors**							
P2Y_12_ receptor	*P2ry12*	3.56	G_i/o_	A	ADP > ATP	↓ cAMP; Platelet aggregation; Microglial migration; Vasoconstriction	Sasaki et al., 2003; Wihlborg et al., 2004
P2Y_14_ receptor	*P2ry14*	1291.7	G_i/o_	A	UDP = UDP-glucose > UDP-galactose > UDP-glucoronic acid > UDP-N-acetyl-glucosamine	↓ cAMP; inflammatory/immune responses	Harden et al., 2010
V_1A_ receptor	*Avpr1a*	1.08	G_q_	A	Vasopressin > oxytocin	↑ IP_3_/DAG; Vasoconstriction	Yang et al., 2010
Y_1_ receptor	*Npy1r*	39.94	G_i/o_	A	Neuropeptide Y = peptide YY > pancreatic polypeptide	↓ cAMP; Inhibits glutamatergic neurotransmission; Vascular remodeling; Vasoconstriction	Crnkovic et al., 2014; Huang and Thathiah, 2015
**Adhesion receptors**							
CELSR2	*Celsr2*	1.03	n.d	Adhesion	Orphan	↑ Ca^2+^; CamKII and Jun kinase activity	Shima et al., 2007; Cortijo et al., 2012; Sugimura et al., 2012
**Frizzled receptors**							
FZD_1_	*Fzd1*	4.86	Canonical Wnt signaling	Frizzled	Wnt-1, Wnt-2, Wnt-3A, Wnt-5A, Wnt-7B	Pericyte motility and polarity during angiogenesis	Nichols et al., 2013; Dijksterhuis et al., 2014; Kilander et al., 2014; Yuan et al., 2015; Corda and Sala, 2017; Henno et al., 2017; Hot et al., 2017; Zimmerli, 2018; Kozielewicz et al., 2020
FZD_3_	*Fzd3*	14.53	G_s_	Frizzled	Wnt-2, Wnt-3A, Wnt-5A	Decoy receptor, dampens Wg signaling	
FZD_6_	*Fzd6*	129.87	G_i/o_, G_q/11_	Frizzled	Wnt-3A, Wnt-4, Wnt-5A, Wnt-5B, Wnt-7A	Cell proliferation, differentiation and polarity	
FZD_7_	*Fzd7*	3.72	G_s_, G_i/o_, Canonical Wnt signaling	Frizzled	Wnt-3, Wnt-3A, Wnt-5A, Wnt-7A	Pericyte motility and polarity during angiogenesis	
FZD_8_	*Fzd8*	4.83	Putative Canonical Wnt signaling	Frizzled	Wnt-2, Wnt-3A, Wnt-9B	n.d	
FZD_10_	*Fzd10*	1.36	Canonical Wnt signaling	Frizzled	Wnt7A, Wnt-7B	Putative role in CNS angiogenesis	
SMO	*Smo*	19.64	G_i/o_, G_12/13_	Frizzled	Constitutively active; oxysterols?	Angiogenesis, remodeling, proliferation and NO release in ECs	
**Orphan receptors**							
GPR4	*Gpr4*	85.06	G_s_, G_i/o_, G_q_, G_12/13_	A	H^+^	↑ cAMP ↑ IP_3_/DAG Pro-inflammatory in ECs	Tobo et al., 2007; Li et al., 2015; Weiß et al., 2017; Carvalho et al., 2020
GPR19	*Gpr19*	15.87	G_s_	A	H^+^	↑ cAMP; Pro-inflammatory in ECs	
GPR20	*Gpr20*	4.93	n.d	A	n.d	n.d	
GPR157	*Gpr157*	1.18	n.d	None	n.d	n.d	
GPR182	*Gpr182*	17.83	n.d	A	Adrenomedullin	n.d	
GPRC5B	*Gprc5b*	1.04	n.d	C	n.d	Regulation of vascular SMC tone	
GPRC5C	*Gprc5c*	385.48	G_12/13_	C	n.d	Reinforces β-catenin and Wnt signaling	
LGR4	*Lgr4*	10.67	Non-classical	A	R-spondin1-4	Implicated role in lipid metabolism	
OPN3	*Opn3*	1.59	n.d	A	n.d	n.d	
TPRA1	*Tpra1*	52.7	G_i/o_	7TM	N/A	n.d	Singh et al., 2015

*The GABA_B_ subunit GABA_B1_ is also expressed by pericytes, but is not included here due to the apparent absence of GABA_B2_, required for functional receptors. Abbreviations not used elsewhere: LPA, lysophosphatidic acid; MMP, matrix metalloproteinase; NAAG, N-acetylaspartylglutamate; PHI, peptide histidine-isoleucine; PHM, peptide histidine-methionine; PHV, peptide histidine-valine; PLD, phospholipase D; TIP39, Tuberoinfundibular peptide of 39 residues; VIP, vasoactive intestinal peptide*.

a *n.d, no data*.

* *Data from He et al. (2018) and Vanlandewijck et al. (2018), expressed as average counts per cell annotated as a brain pericyte. Cells were isolated from adult mice of either sex aged 10–19 weeks*.

** *We note here the principal transduction G-protein, although many receptors are promiscuous and couple to secondary transduction pathways. Frizzled receptors canonically couple to Wnt signaling but may also interact with a rage of G proteins. Where there is no clear primary pathway, we list all possibilities. Readers are referred to Alexander et al. (2019) for further details*.

**Figure 1:**
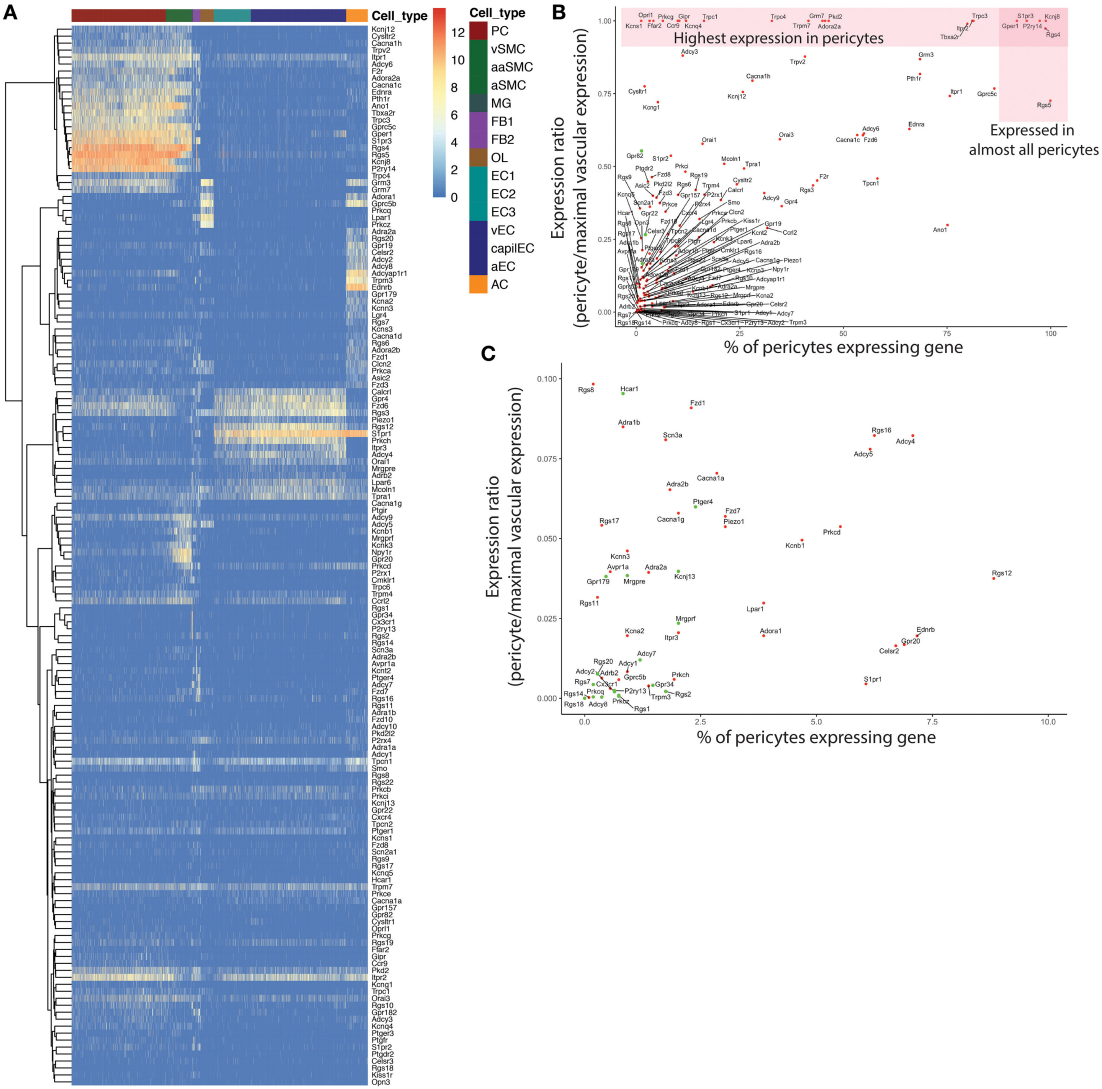
Overview of gene qualification process for pericyte ion channels and GPCRs and other genes of interest. An initial filter of 1 average count/cell was applied to exclude genes with extremely low expression. **(A)** Heatmap of expression of the remaining genes throughout the neurovascular unit. A small subset of these genes were highly enriched in pericytes (*top left*), while many showed higher expression in other cell types. To filter out potential contamination, genes that were expressed in <3% of pericytes, and were absent from the PER3 cluster of Zeisel et al. (2018) were excluded. **(B)** Relationship between pericyte-specificity of expression and fraction of pericytes expressing each gene considered. Genes represented by green circles were excluded according to the above criteria. **(C)** High resolution view of genes with a <0.1 expression ratio in pericytes, that were expressed in fewer than 10% of pericytes, corresponding to the bottom left corner in **(B)**. Genes represented by green circles were excluded from further consideration as potential contamination.

An important caveat with this approach is that mRNA expression does not necessarily predict protein levels (Liu et al., 2016), and we thus stress that it is essential that the hypotheses generated by transcriptomic data be subject to further experimental scrutiny. Accordingly, while the following discussion is based on robust mRNA expression data, we highlight where there is question of whether gene expression translates into functional channels or receptors. A second putative caveat relates to the quality of the scRNAseq data. Specifically, it is important to ask if low-level mRNA counts reflect true and physiologically meaningful expression or artifacts such as contamination of the pericyte transcriptomes by mRNA from other cell types. Pericytes in particular are sensitive to endothelial contamination because of the tight physical association between these two cell types. With these caveats in mind, to arrive at a list of genes with reasonable likelihood of pericyte expression we first selected genes detected at levels >1 average count per cell in the 1,088 adult brain pericytes present in the Vanlandewijck et al. dataset (http://betsholtzlab.org/VascularSingleCells/database.html; He et al., 2018; Vanlandewijck et al., 2018) and compared this to their expression in the Zeisel dataset (http://mousebrain.org; Zeisel et al., 2018). In the latter, three pericyte clusters are provided (PER1, PER2, PER3) of which PER1 and PER2 are endothelial cell contaminated, whereas PER3 appears pure. After manually checking for signs of contamination by comparing the expression level in pericytes with expression in other brain cell types, we selected the following criteria as qualifying: (i) expression in >3% of the pericytes in the Vanlandewijck dataset and; (ii) detectable expression (>0) in the Zeisel et al. PER3 dataset (Figure 1).

Below, we focus our discussion on the ion channels and GPCRs that are likely to be most pertinent to blood flow control. We center our discussion on studies using acute and *in vivo* preparations, as cultured pericytes may exhibit phenotypic drift which confounds interpretation. Accordingly, we note instances in which we refer to cultured pericytes. We begin by briefly reviewing the key features of the brain vasculature and pericytes before exploring their ion channel and GPCR complement in detail.

## The Vascular Network of the Brain

### Fundamental Angioarchitecture

**Figure 2:**
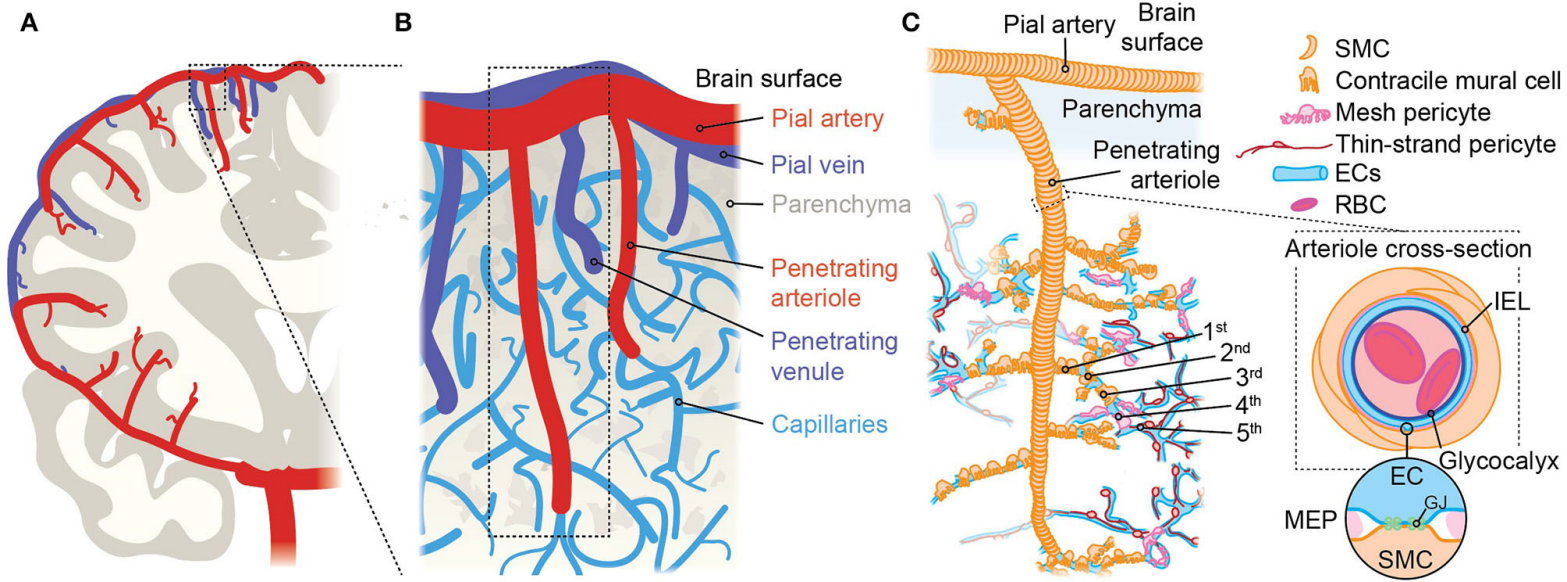
An overview of brain angioarchitecture. **(A)** Cross-section of one brain hemisphere illustrating macroscopic vascular architecture. The carotid artery joins the circle of Willis at the base of the brain, then gives rise to major pial arteries which course over the brain surface, from which multiple penetrating arterioles arise and dive into the tissue. **(B)** Close up view of the components of the vascular network approximating the area in the boxed region in *A* showing the interconnected organization of pial arteries, penetrating arterioles, the dense capillary network, and venules. The vessel labeling system we use takes the penetrating arteriole as the 0-order vessel and primary reference point, and vessels are numbered sequentially with regard to this. Vessel number automatically increases each time a vessel branches and thus, after vessel *n* branches, the daughter branches—regardless of diameter or orientation—are labeled vessel *n* + 1. **(C)** Illustration approximating the boxed region in **(B)**, showing the cellular elements that make up the arteriolar side of the brain vasculature. Arteries and arterioles consist of SMCs surrounding ECs, which are in direct contact with the blood. The first 3–4 vessels emanating from the penetrating arteriole are a transitional zone and are covered with contractile mural cells that are positive for α-SMA and can change diameter abruptly. Immediately after the α-actin terminus are capillaries covered by mesh pericytes, following which are capillaries where thin-strand pericytes reside. The cross-section at right shows a section through an artery/arteriole and illustrates the presence of the internal elastic lamina (IEL) which separates ECs and SMCs. Occasional fenestrations dot the IEL, through which ECs and SMCs make direct contact via myoendothelial projections (MEPs, *circular inset*). These are sites of gap junctions (GJs) permitting chemical and electrical cell-cell communication.

From pial arteries on the brains surface, penetrating arterioles branch orthogonally and dive into the parenchyma (Duvernoy et al., 1981; Cipolla, 2009; Figure 2). Arteries and arterioles are composed of a lumen lined by electrically-coupled cobblestone–morphology ECs (Haas and Duling, 1997) that directly interface with the blood. These ECs are surrounded by a fenestrated internal elastic lamina (IEL), composed mainly of elastin and collagen (Schwartz et al., 1981), through which they extend projections to directly contact overlying contractile smooth muscle cells (SMCs) (Aydin et al., 1991).

As the penetrating arteriole extends deeper into the tissue, further vessels sprout from its length at regular intervals (Blinder et al., 2013). These initial branch points are sites of precapillary sphincters which are regulated over short time scales to control blood flowing into the capillary bed (Grubb et al., 2020). From this point, extensive ramification of the vascular bed greatly expands the surface area of the network, facilitating efficient exchange of nutrients and waste to rapidly satisfy the intense metabolic requirements of every neuron. The capillary bed—consisting of capillary ECs (cECs; Garcia and Longden, 2020) and overlying pericytes (see below) embedded in the basement membrane (a dense network of glycoproteins, collagens and secreted factors; Pozzi et al., 2017)—is incredibly dense, and each microliter of cortex holds approximately 1 m of blood vessels (Shih et al., 2015). Of these, around 90% by volume are capillaries (Gould et al., 2017). Accordingly, ECs are estimated to comprise around 30% of the non-neuronal cell mass in the gray matter, forming a network of 20–25 billion ECs throughout the entire human brain (von Bartheld et al., 2016). This places cECs in close apposition with all neurons, with each neuronal cell body lying within ~15 μm of a vessel (Tsai et al., 2009). Red blood cells (RBCs) traverse this network, releasing oxygen to diffuse down its concentration gradient into the tissue, while glucose is transported by ECs from the blood plasma into the parenchyma. After negotiating the capillary bed, oxygen-depleted RBCs eventually reach a vertically-oriented venule, which drain to veins at the cortical surface on the path back to the heart.

### Mural Cell Properties Transition Gradually With Increasing Branch Order

**Figure 3:**
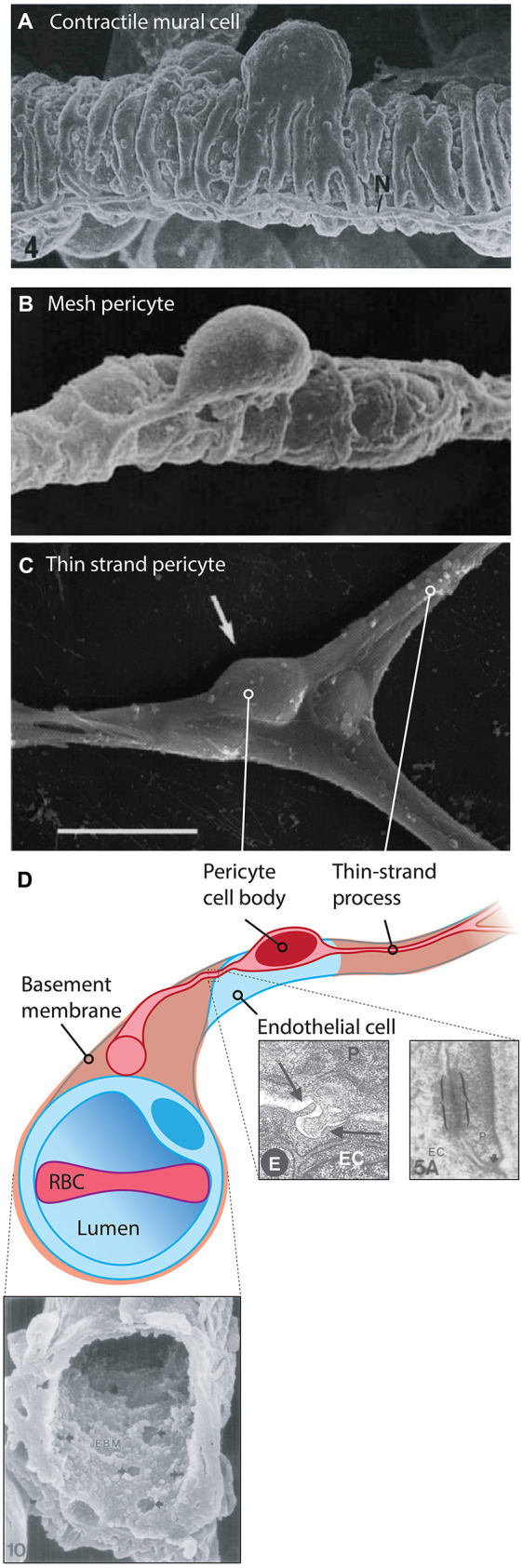
Cytoarchitecture and microenvironment of pericytes. **(A)** Mural cells with a ‘bump-on-a-log’ cell body, with multiple contractile processes that almost completely encase the underlying vessel. 6,000x, rat mammary gland vasculature. Reproduced with permission from Fujiwara and Uehara (1984). **(B)** A 4,400x magnification scanning electron micrograph of a putative mesh pericyte of the rat mammary gland. Multiple sparse processes enwrap the underlying capillary. Reproduced with permission from Fujiwara and Uehara (1984). **(C)** A thin-strand pericyte atop a rat retinal capillary, extending fine processes away from the ovoid cell body. Adapted with permission from Sakagami et al. (1999). Scale bar: 10 μm. **(D)** Illustration of a thin-strand pericyte. The bulk of the volume of the cell body is occupied by the nucleus. The pericyte is prevented from making direct contact with the underlying EC by the basement membrane, shown in the SEM at *bottom left*, reproduced with permission from Carlson (1989). Multiple small fenestrations are seen in this structure, allowing for pericyte and endothelial projections to make direct contact with one another, forming so-called ‘peg-socket junctions’ which are also sites of gap junction formation. At *bottom right* electron micrographs depicting a peg-socket junction (left) and a pericyte-endothelial gap junction (right) are shown, reproduced with permission from Díaz-Flores et al. (2009) and Carlson (1989). Abbreviations in micrographs: EC, endothelial cell; N, nerve; P, pericyte.

As the vascular bed ramifies from the penetrating arteriole, there is gradation in the morphology and functional characteristics of the mural cells associated with vessels. The first 3–4 branches of the vascular network (1st to 4th order) originating from the penetrating arteriole constitute a “transitional zone” (Ratelade et al., 2020). These vessels are covered by cells expressing high levels of α-smooth muscle actin (α-SMA) with ovoid cell bodies and multiple broad processes that almost completely ensheathe the underlying vessel (Grant et al., 2019; Figure 3A). Given that the identity of these cells is unresolved, and that they have been referred to as both pericytes (Peppiatt et al., 2006; Hall et al., 2014; Attwell et al., 2016; Grant et al., 2019) and SMCs (Hill et al., 2015; Grutzendler and Nedergaard, 2019), we refer to these cells here as “contractile mural cells” and to the segments of the vasculature that they cover as “vessels.” Expression of α-SMA permits these cells to rapidly regulate the diameter of the underlying vessel and therefore blood flow. Indeed, multiple studies have illustrated the importance of contractile mural cells in mediating dilation (of ~10–30%) in response to neuronal stimulation (Hill et al., 2015; Mishra et al., 2016; Kisler et al., 2017; Cai et al., 2018; Rungta et al., 2018).

Beyond this point in the vasculature, mural cells do not express high levels of α-SMA, although one recent study suggested that retinal mural cells retain expression of a low level of this protein (Alarcon-Martinez et al., 2018) and they do express very low levels of the *Acta2* gene in the brain (He et al., 2018; Vanlandewijck et al., 2018). As a result, these cells are not equipped to regulate vessel diameter over abrupt time scales, but there is clear evidence that they may contract slowly under certain circumstances (reducing the diameter of the underlying vessel by up to ~25%; Fernández-Klett et al., 2010; Gonzales et al., 2020). Thus, we consider the relatively static diameter vessels downstream of the α-SMA terminus (which typically occurs between the 1st and 4th order branch in immunostaining experiments; Grant et al., 2019) to be capillaries. The identity of mural cells on these so-defined capillaries is unambiguous, and there is consensus that these cells are pericytes.

The pericytes residing on capillaries display at least two distinct morphologies: (i) Immediately adjacent to the α-SMA terminus, pericytes take on a mesh-like appearance, and are thus known as “mesh pericytes” (Figure 3B); (ii) beyond these are cells that project long, thin processes along the vasculature, and accordingly these are referred to as “thin-strand pericytes” (Grant et al., 2019; Figures 3C,D).

## Cellular Anatomy of Mesh and Thin-Strand Pericytes

Despite differing morphologies (Figure 3), mesh and thin-strand pericytes are indistinguishable at the level of single-cell transcriptomics, possibly due to the fact that mesh pericytes represent only a small fraction of capillary pericytes (Chasseigneaux et al., 2018). Pericyte cell bodies have a highly stereotyped shape, appearing as a large ovoid that protrudes from the wall of the capillary, which is often referred to as a “bump-on-a-log” (Grant et al., 2019). Mesh pericytes are few in number relative to thin-strand pericytes and have fewer, shorter longitudinal processes (their primary trunks averaging 40 μm in length; Hartmann et al., 2015) that cover ~70% of the underlying capillary. This contrasts with upstream contractile mural cells which cover 95% of the underlying vessel (Grant et al., 2019). Thin-strand pericytes extend long, thin, strand-like processes that are ~1.5 μm in diameter and cover on average around 250 μm in total capillary distance, in some instances exceeding 300 μm (Berthiaume et al., 2018). Together, the thin-strand pericyte cell body and its processes cover between one third (Mathiisen et al., 2010) and one half (Grant et al., 2019) of the abluminal surface area of the endothelium. A typical thin-strand process has a stable “non-terminal core” of ~50 μm in length that bifurcates into slightly shorter, dynamic terminal processes that may extend or retract up to 20 μm over the course of days to weeks (Berthiaume et al., 2018). At their terminal ends, thin-strand processes appear to come into close proximity with those of neighboring pericytes (Berthiaume et al., 2018), possibly allowing for direct contact between adjacent pericytes, although this awaits direct experimental confirmation. Changes in the length of processes of one cell appear to evoke opposite changes in the length of adjacent pericyte processes, preventing the formation of substantial gaps (Berthiaume et al., 2018).

These processes are for the most part prevented from making direct contact with the underlying endothelium by the basement membrane. However, electron microscopy has revealed that—similar to the IEL of arteries and arterioles—the capillary basement membrane is dotted with many fenestrations, with an average area of 1.5 μm^2^, ranging from 100 to 450 nm in diameter (Carlson, 1989; Figure 3D). In arteries, similar fenestrations are the sites of myoendothelial junctions, optimized for EC-SMC communication by the presence of a number of key enzymes, ion channels, and gap junction (GJ) proteins (Straub et al., 2014). In the capillary bed, these fenestrations are the site of “peg-socket” interdigitations where either the pericyte or the EC sends a projection to make contact with the adjacent cell (Tilton et al., 1979; Cuevas et al., 1984; Armulik et al., 2005). These contact points are thought to be the sites of GJ communication between the two cell types (see Box 1), and may be the location of key signaling events, such as local calcium (Ca^2+^) or cyclic adenosine monophosphate (cAMP) elevations. Moreover, they may be sites of macromolecular signaling complex assembly, containing ion channels, and GPCRs positioned to facilitate cell-cell communication.

Box 1 **Potential gap junction configurations between capillary pericytes and cECs.**

According to expression data (He et al., 2018; Vanlandewijck et al., 2018), pericytes predominantly express mRNA for connexin (Cx)37 and Cx45, along with much lower expression of Cx26 and Cx43. Capillary ECs, on the other hand, robustly express Cx43 and Cx45, with low levels of Cx37, whereas Cx26 is undetectable (see Figure). Electron microscopy has been used to visualize putative GJ sites between pericytes and ECs at peg-socket interdigitations. In contrast, similar sites between the processes of neighboring pericytes have yet to be clearly demonstrated. Nonetheless, a recent dye transfer study (Kovacs-Oller et al., 2020), has shown that the cells of the capillary bed form a syncytium. Accordingly, two configurations for cell-cell communication can be postulated: (i) Pericyte-EC GJs alone permit bidirectional transfer of intracellular materials and charge between cells of the capillary wall; (ii) both pericyte-EC GJs and pericyte-pericyte GJs permit intercellular communication along two parallel, closely adjacent paths. The latter configuration would provide redundancy in the event of cell-cell communication failing in one cell type.

GJs are homo- or hetero-dodecameric assemblies of Cx subunits (Koval et al., 2014), formed from two hexameric hemichannels that dock to yield intercellular channels. GJs can be homotypic, with both hemichannels composed of the same Cx isoform(s), or heterotypic, with each hemichannel consisting of a distinct assembly of 6 Cx subunits. Moreover, a given hemichannel may be homomeric (composed Cx monomers of the same isoform) or heteromeric (consisting of multiple Cx isoforms), a property that depends on the propensity of the locally expressed Cxs to co-assemble. These complexities yield channels with distinct attributes, which may further oligomerize into large GJ plaques with discrete population characteristics.

Considering pericyte connexins in isolation, α-class Cxs 37 and 45 are not known to assemble into heteromers, but both of these will heteromerize with the much more modestly expressed α Cx43. The β Cx26, on the other hand, is not compatible with α Cx isoforms. Thus, the available data suggest that the typical pericyte hemichannel is most likely to be a homomeric assembly of Cx37 or Cx45, with perhaps a low level of heteromerization involving Cx43. Similarly, the EC-expressed Cx43 will form heteromers with Cx37 and Cx45, but again the latter are not compatible with one another. Thus, the possibility of heteromerization appears to be higher for ECs. In terms of heterotypic compatibility in the formation of GJs, Cx37, Cx43, and Cx45 are known to readily assemble together, whereas Cx26 hemichannels will not dock with any of these.

**Figure:**
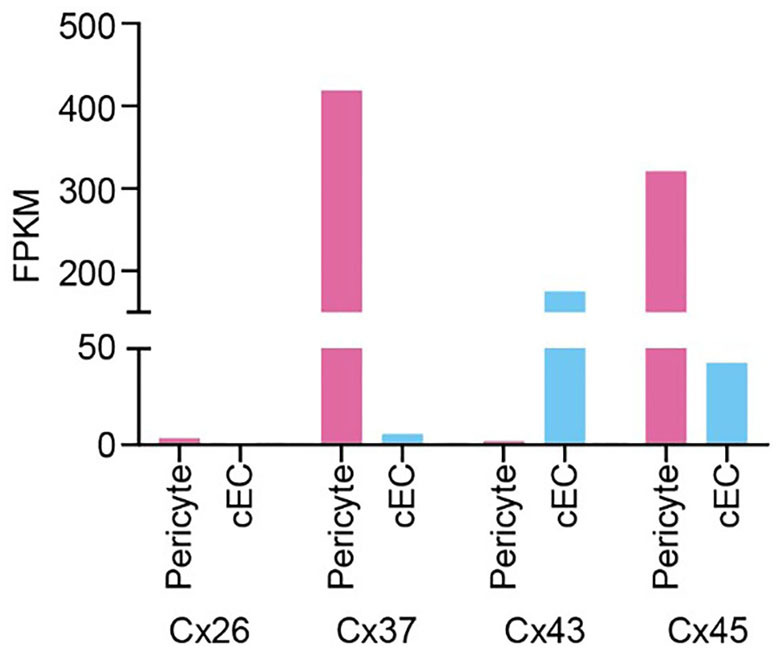

Taken together, this complexity underscores the great deal of further work needed to firmly establish the nature and properties of GJs in the capillary wall.

## Ion Channel Expression in Brain Capillary Pericytes

**Figure 4:**
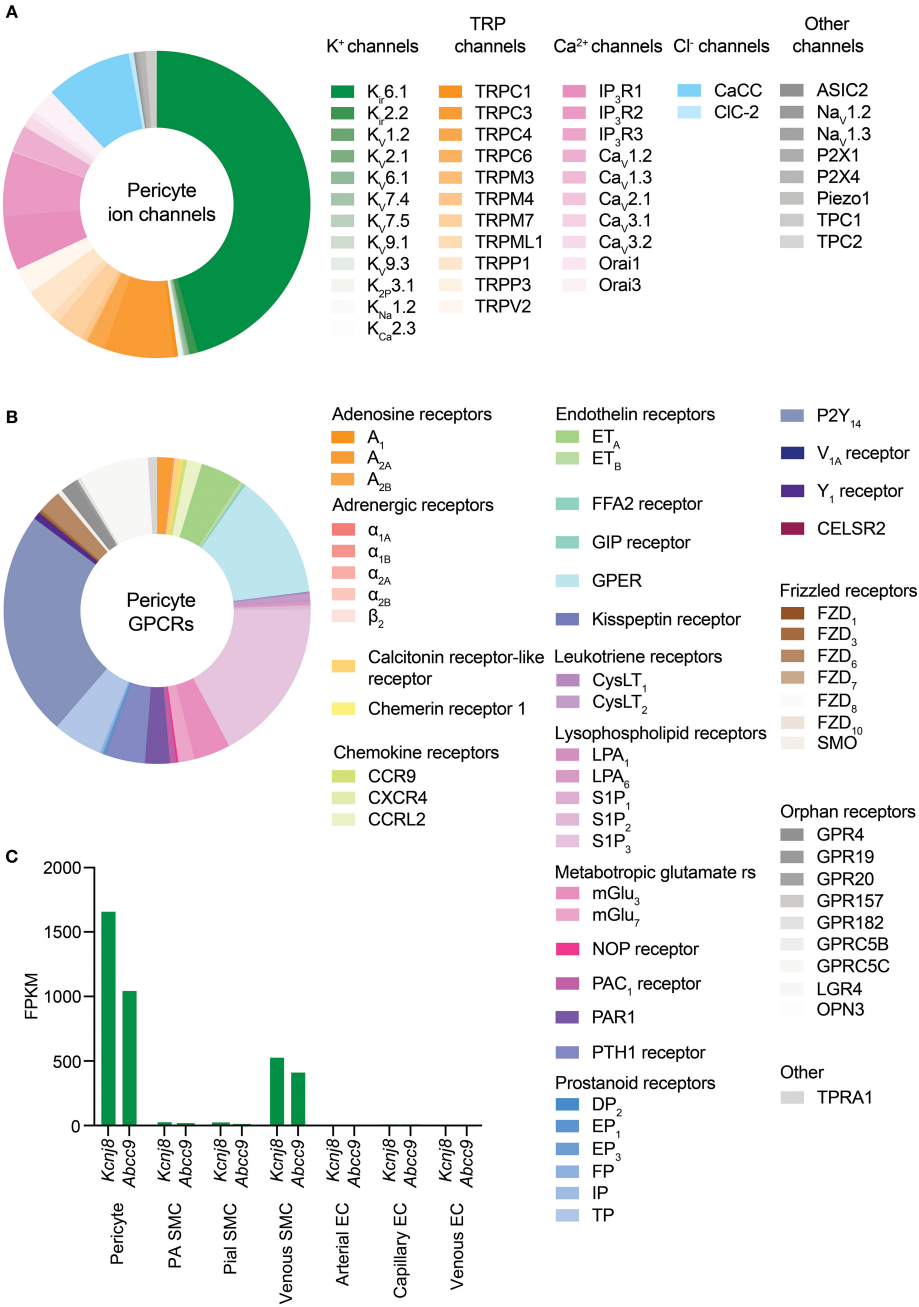
Overview of CNS pericyte ion channel and GPCR expression. **(A)** Relative abundance of mRNA for all ion channel subunits meeting our inclusion criteria. The size of each segment represents the relative expression of the underlying gene. Channels are clustered on the basis of the ion species that the corresponding functional channel conducts (denoted by shading of the same color) and are then grouped by family/subfamily. K^+^ channels are the predominant ion channel class due to extremely high expression of *Kcnj8* which forms the pore of vascular K_ATP_ channels. The non-selective TRP channels are the next highest expressed, followed by Ca^2+^ channels, Cl^−^ channels, and lower expression of other channels. **(B)** Relative expression of pericyte GPCRs. Here, receptors are organized by ligand sensitivity or class. **(C)** Expression of the K_ATP_ channel genes *Kcnj8* and *Abcc9* throughout the brain vasculature. Pericytes express both genes at much higher levels than arterial SMCs or ECs. However, venous SMCs also express high levels of K_ATP_ channel-forming genes.

A cursory review of the brain capillary pericyte ion channel expression data provided by He et al. (2018) and Vanlandewijck et al. (2018) reveals that potassium (K^+^) channels are the dominant ion channel species in pericytes. Remarkably, this is due to the adenosine triphosphate (ATP)-sensitive K^+^ (K_ATP_) channel inward rectifier (K_ir_) subunit, K_ir_6.1, accounting for nearly half of the total ion channel gene expression in these cells. Transient receptor potential (TRP), Ca^2+^, and chloride (Cl^−^) channels make up the remaining half, along with lower expression of a handful of other channel subunits including two-pore channels (TPCs), voltage-gated sodium (Na^+^; Na_v_) channels, P2X receptors, acid sensing ion channels (ASICs), and Piezo1 (Table 1 and Figure 4).

## Pericyte K^+^ Channels

Focusing initially on the K^+^ channel superfamily, capillary pericytes express K_ir_, two-pore domain (K_2P_), voltage-gated (K_v_), Na^+^-activated (K_Na_), and Ca^2+^-activated (K_Ca_) K^+^ channel genes.

### K_ir_-Family Channels May Enable Pericyte Metabolism-Electrical Coupling and Facilitate Rapid, Long-Range Electrical Signaling

K_ir_ channels have the defining biophysical property of inward rectification, preferentially conducting large currents into the cell at voltages negative to the K^+^ equilibrium potential (E_K_), the magnitude of which depend on the electrochemical gradient for K^+^ [i.e., the difference between V_m_ and E_K_] (Katz, 1949; Hibino et al., 2010). At potentials positive to E_K_ some degree of rectification occurs, ranging from strong—in which almost no current passes from the interior of the cell to the exterior—to weak, in which rectification is only seen at very positive potentials. Accordingly, K_ir_ channels can be classified by their degree of rectification as strongly-rectifying (K_ir_2.x, K_ir_3.x), intermediately-rectifying (K_ir_4.x) or weakly-rectifying (K_ir_1.1, K_ir_6.x, K_ir_7.x). Alternatively, this group of channels can be classified according to function into classic (K_ir_2.x), G-protein sensitive (K_ir_3.x), K_ATP_ (K_ir_6.x), or K^+^ transport (K_ir_1.x, K_ir_4.x, K_ir_5.x, K_ir_7.x) channels (Hibino et al., 2010). Of the K_ir_ channel family, capillary pericytes express extremely high levels of K_ir_6.1—far exceeding that of any other ion channel gene expressed by brain pericytes—and to a lesser extent K_ir_2.2 (Bondjers et al., 2006; He et al., 2018; Vanlandewijck et al., 2018).

**Figure 5:**
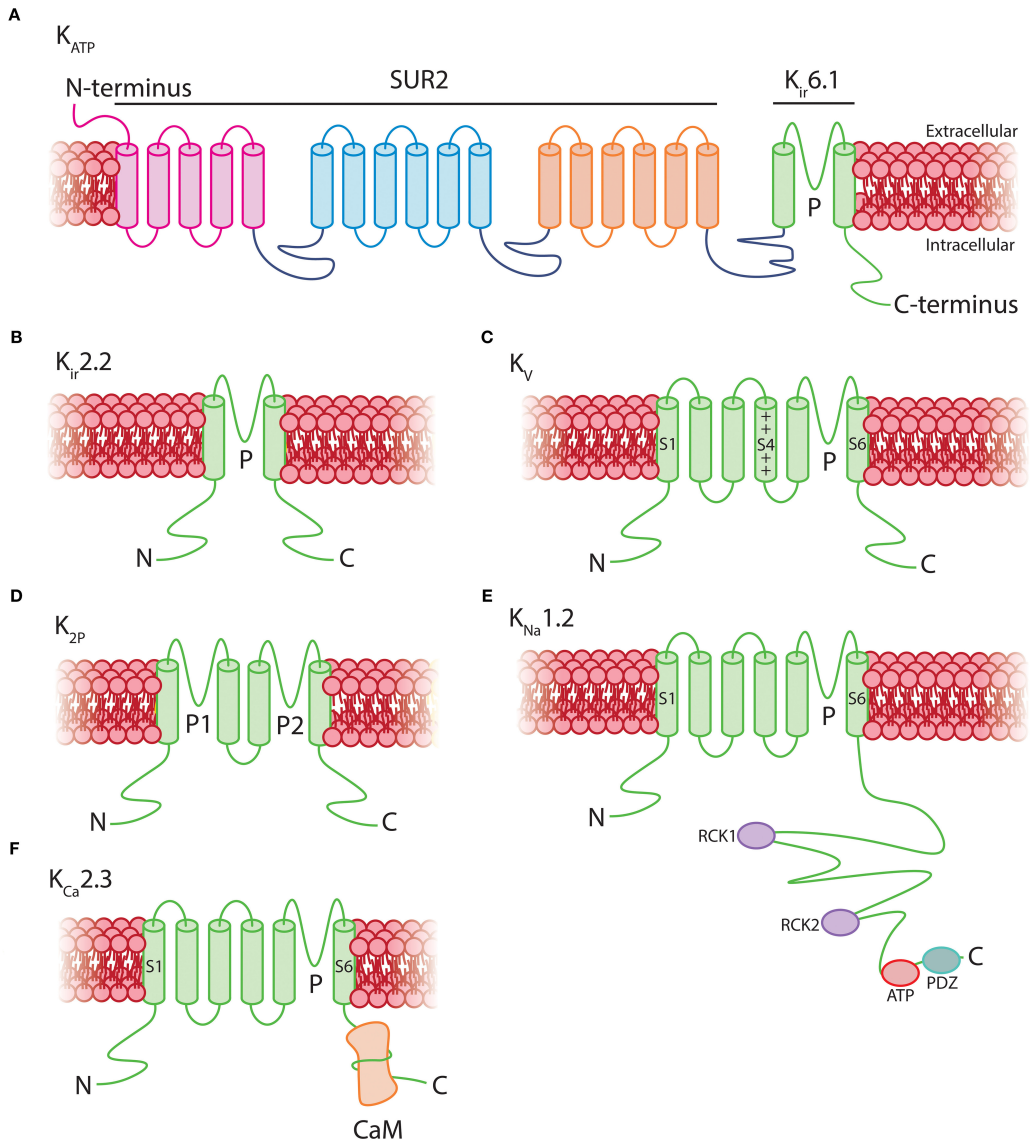
Structural topology of K^+^ channels expressed by pericytes. **(A)** Vascular K_ATP_ channels are octamers consisting of four 17-transmembrane SUR2 subunits associated with four 2-transmembrane pore-forming K_ir_6.1 subunits. **(B)** K_ir_2.2 channels consist of homo or heteromeric assemblies of four 2-transmembrane subunits. **(C)** K_v_ channels are composed of four 6-transmembrane alpha subunits with a positively charged voltage sensor at S4 which transduces changes in V_m_ into conformational alterations. **(D)** K_2P_ channels are tetramers of two-pore domain four-transmembrane subunits. **(E)** K_Na_ channels have a 6-transmembrane structure that lacks a voltage sensor, with multiple regulatory sites in the long intracellular COOH-terminus including two RCK domains, an ATP binding site, and a PDZ domain. **(F)** K_Ca_2.3 channels consist of four 6-transmembrane domains which lack a voltage-sensor at S4. The COOH-terminus of each is associated with a calmodulin monomer, which imparts Ca^2+^ sensitivity to the channel.

As K_ir_6.1 is a component of K_ATP_ channels, this suggests that the two key roles of these channels—providing membrane hyperpolarization and coupling metabolism to membrane electrical activity—could be major contributors to pericyte physiology. Functional K_ATP_ channels are hetero-octameric assemblies of four two-transmembrane spanning pore–forming K_ir_6.x subunits (either K_ir_6.1 or K_ir_6.2, encoded by *Kcnj8* and *Kcnj11*, respectively), each associated with a regulatory 17-transmembrane spanning ATP-binding cassette subfamily sulfonylurea subunit (SUR1 or SUR2, respectively encoded by *Abcc8* and *Abcc9*—the latter of which is also highly expressed in brain pericytes; Figure 5A; Seino and Miki, 2003; Li et al., 2017). SURs are required for membrane trafficking of the channel (Burke et al., 2008) and impart sensitivity to K_ATP_ agonists and antagonists and intracellular nucleotides. Alternative splicing yields a number of SUR2 variants with SUR2A and SUR2B as the major forms, differing by just 42 amino acids in their C-terminal domains (Seino and Miki, 2003). Thus, the available expression data suggest that K_ATP_ channels native to brain pericytes are composed of K_ir_6.1 and SUR2—often referred to as the “vascular” form of K_ATP_–and indicates that these are expressed much more highly in pericytes than they are in cerebral SMCs and ECs (Figure 4C).

K^+^ currents through K_ATP_ channels are weakly rectifying at potentials very positive to E_K_–the result of voltage-dependent intracellular magnesium (Mg^2+^) block (Findlay, 1987). The defining biophysical feature of K_ATP_ channels is that open probability (P_o_) decreases with increasing intracellular ATP levels, with ATP stabilizing the closed state of the channel (Enkvetchakul and Nichols, 2003). Thus, when cellular ATP demands are low and free cytosolic ATP is high, the channel is closed. In contrast, when cell activity increases or metabolism drops, the ADP:ATP ratio rises and the channel may open to hyperpolarize the membrane (Quayle et al., 1997). Consistent with these channels being saturated by ATP to keep them closed under resting conditions, the K_ATP_ channel blocker glibenclamide has no effects on resting CBF but levcromakalim, a K_ATP_ channel opener, increases global CBF by 14% (Al-Karagholi et al., 2020).

Nucleotide regulation of K_ATP_ channels is complex and has been best characterized for K_ir_6.2/SUR1-containing channels, which we review briefly here. Intracellular nucleotides are sensed by an array of sites throughout the channel complex: ATP has been shown to bind to an inhibitory site of the K_ir_6.2 subunit (Tucker et al., 1997; Tanabe et al., 2000) with just one of four subunits of the channel needing to bind ATP to effect closure (Markworth et al., 2000). The SUR1 subunit has two nucleotide binding domains (Li et al., 2017), where Mg^2+^-bound adenosine diphosphate (MgADP) occupancy increases channel activity (Tung and Kurachi, 1991; Gribble et al., 1997; Shyng et al., 1997). MgATP also has a stimulatory effect here, likely through hydrolysis to MgADP, although this is normally masked by the much more potent inhibitory effect of free ATP (Gribble et al., 1998; Proks et al., 2010). Thus, as might be expected, increasing intracellular Mg^2+^ antagonizes the inhibitory effect of free ATP (Gribble et al., 1998). Conversely, in the absence of Mg^2+^, ADP may have an inhibitory effect (Findlay, 1988). Comparatively less is known about the fine details of nucleotide regulation of K_ir_6.1/SUR2B channels, which have a smaller conductance than their K_ir_6.2-containing counterparts (~15–30 pS for K_ir_6.1/SUR2B-containing channels vs. ~50–90 pS for the K_ir_6.2/SUR2A form, for example; Hibino et al., 2010). However, it is clear that the presence of a nucleotide diphosphate and Mg^2+^ is a requirement for channel activity, and that these channels are also sensitive to ATP inhibition (Kajioka et al., 1991; Kovacs and Nelson, 1991; Beech et al., 1993; Kamouchi and Kitamura, 1994; Nelson and Quayle, 1995; Zhang and Bolton, 1996; Yamada et al., 1997).

One of the consequences of the nucleotide sensitivity of K_ATP_ channels is that they may act as sensors of the metabolic state of the cell and transduce changes in this parameter into adjustments of membrane voltage. This is perhaps best characterized in pancreatic β cells, where K_ATP_ channels composed of K_ir_6.2 and SUR1 subunits couple glucose concentration with insulin secretion (Tarasov et al., 2004). Here, elevated glucose leads to an increase in intracellular ATP due to increased glucose metabolism. This closes K_ATP_ channels, which depolarizes the cell and drives Ca^2+^-mediated insulin secretion through the activation of L-type voltage-dependent Ca^2+^ channels (VDCCs; MacDonald et al., 2005). Conversely, if glucose concentrations decrease the channel opens, hyperpolarizing the membrane to prevent insulin release. In an analogous situation, K_ATP_ channels composed of K_ir_6.2 and SUR1 are involved in glucose sensing and glucagon secretion in the ventromedial hypothalamic neurons of the hypothalamus (Miki et al., 2001).

Like many other channels (Hille et al., 2015; Dickson and Hille, 2019), K_ATP_ channels containing K_ir_6.2 pore-forming subunits are also influenced by the concentration of intracellular phosphoinositides, such as phosphoinositol-4,5-bisphosphate (PIP_2_; Fan and Makielski, 1997). In K_ir_6.2-containing channels, ATP and PIP_2_ compete for residues on overlapping binding sites on the pore forming subunit, each subtly altering channel conformation to stabilize closed or open states, respectively (Enkvetchakul and Nichols, 2003), with PIP_2_ additionally uncoupling the pore-forming subunit from its SUR companion (Li et al., 2017). Exposure of these K_ATP_ channels to PIP_2_ decreases ATP affinity (K_0.5_) in excess of two orders of magnitude from ~10 μM to ~3.5 mM, and furthermore in the absence of ATP increases channel P_o_ (Shyng and Nichols, 1998). As the abundance of PIP_2_ thus regulates P_o_, this raises the possibility that cell signaling that impinges upon PIP_2_ levels may subsequently affect channel activity. K_ir_6.1/SUR2B channels, in contrast, appear to have a much higher affinity for PIP_2_ than K_ir_6.2 channels. Accordingly, PIP_2_ is thought to bind so tightly here as to be saturating, and thus physiological fluctuations of this phospholipid do not influence channel activity (Quinn et al., 2003; Harraz et al., 2020). However, a number of intracellular signaling pathways have been established to dramatically influence vascular K_ATP_ activity. Indeed, phosphorylation by protein kinase C (PKC), lying downstream of DAG, decreases the P_o_ of K_ir_6.1/SUR2B channels (Bonev and Nelson, 1996; Shi et al., 2008b) and in stark contrast, protein kinase A (PKA), which is stimulated as a result of G_s_-coupled GPCR engagement, phosphorylates K_ATP_ to increase P_o_ (Kleppisch and Nelson, 1995; Bonev and Nelson, 1996; Quinn et al., 2004; Shi et al., 2007, 2008a).

Accordingly, there appear to be two major possible avenues through which vascular K_ATP_ channels could be engaged in pericytes:

*i) Changes in metabolism may couple K*_*ATP*_ *channel activity to membrane hyperpolarization*.

It is possible that brain pericyte K_ATP_ channels act as sensors of the metabolic state of the cell and adjust membrane potential in response to perturbations in energy supply. Notably, the expression of the glucose transporter GLUT1 is incredibly high in astrocytes and brain ECs compared to pericytes, which express much lower levels of GLUTs 1, 3 and 4 (He et al., 2018; Vanlandewijck et al., 2018). Therefore, while astrocytes and capillary endothelial cells are well equipped for glucose import, the comparatively lower expression of GLUTs in the pericytes situated between them could make them more sensitive to subtle changes in glucose levels, such as local depletions that occur during neural activity (Hu and Wilson, 1997; Paulson et al., 2010; Li and Freeman, 2015; Pearson-Leary and McNay, 2016). Such decreases in glucose could impact pericyte metabolism, increasing the ADP:ATP ratio to open K_ATP_ channels and hyperpolarize the membrane.

However, as glucose can be transmitted via gap junctions (Rouach et al., 2008) it is possible that pericyte glucose needs are instead satisfied directly by the underlying ECs, enabling them to continually maintain a high level of cytosolic ATP. This latter possibility, coupled with evidence that metabolic regulation of vascular K_ATP_ channels in arteriolar SMCs requires either anoxia or extreme ATP consumption (Quayle et al., 2006)—circumstances of energetic compromise that are unlikely to be seen under physiological conditions (Quayle et al., 1997)—suggests that K_ATP_ metabolism-electrical coupling may be primarily relevant in pathological situations (e.g., stroke). In this context, metabo-electrical coupling may represent a last-ditch effort to stimulate blood flow and therefore replenish O_2_ and glucose to regions in deep metabolic crisis. Further studies are needed to understand metabolic contributions to the control of pericyte K_ATP_ channels.

*ii) Molecules that stimulate G*_*s*_ *signaling may engage pericyte K*_*ATP*_ *channels*.

Pericytes express a broad repertoire of receptors that couple to the G_s_ signaling pathway, including those for purines, polyadenylate cyclase activating peptide (PACAP), parathyroid hormone (PTH) and prostaglandins (discussed in detail below, see Table 2). The release of these molecules into the paravascular space during neuronal activity could thus engage G_s_ signaling in local pericytes, culminating in the phosphorylation of K_ATP_ and channel opening. Indeed, in the retina (often used as a model of the NVU; see Box 2) the inhibitory neurotransmitter and metabolic byproduct adenosine hyperpolarizes the rat retinal pericyte membrane potential by ~30 mV through K_ATP_ channel engagement resulting from A_1_ and A_2a_ adenosine receptor activation (Li and Puro, 2001), likely through engagement of cAMP and PKA.

Box 2 **A brief comparison of retinal and brain vasculatures.**

The retinal vasculature consists of two vascular beds—the outer layer of retinal photoreceptors is nourished by the choroidal vasculature, and the multilayered inner retinal vasculature provides oxygen and nutrients to the inner cell layers. The latter has a tightly regulated blood-retinal barrier, akin to the BBB, which pericytes help to maintain (Trost et al., 2016). Vascular density in the cerebral cortex varies according to the metabolic demand of the brain region it supplies (e.g., white vs. gray matter), whereas in the retina, capillary density tends to be greater in the center of the tissue and decreases toward the periphery (Patton et al., 2005). Both retinal and cerebral vascular cells have identical embryological origins: pericytes and SMCs derive from neuroectodermal neural crest cells and ECs derive from mesodermal hemangioblasts (Kurz, 2009; Dyer and Patterson, 2010). Structurally, the cortical and inner retinal vascular beds share a similar overall architecture, with a post-arteriolar transitional zone of 3–4 branches that are covered by contractile mural cells, leading to thin strand pericyte-covered deep capillaries (Ratelade et al., 2020). A distinction between these vascular beds is that the retinal vasculature is highly organized into two parallel plexi (Ramos et al., 2013), whereas cerebral capillaries form more elaborate three-dimensional geometries (Blinder et al., 2013). These structural differences could dictate differences in the flow of blood through each circulation and may necessitate distinctions in the signaling mechanisms that are utilized to direct blood flow through either bed. However, the vasculatures in both retina and cortex respond similarly to neuronal activity with elevations in blood flow (Newman, 2013), and similar mechanisms underpinning these responses appear to be at play in either bed. K^+^, PGE_2_, and EETs, for example, have been implicated in control of blood flow in both circulations (Newman, 2013; Longden et al., 2017; Gonzales et al., 2020). Recent studies have also indicated the utility of non-invasive examinations of the retinal vasculature as a marker for detecting cerebrovascular diseases, due to a similar susceptibility of both circulations to vascular risk factors such as hypertension or diabetes (Patton et al., 2005; van de Kreeke et al., 2018; McGrory et al., 2019; Querques et al., 2019). Data on gene expression in vascular cells of the retina are currently lacking, but would provide a useful standpoint for deeper comparisons of the similarities and differences between these vascular beds.

Studies on retinal pericytes (Li and Puro, 2001; Kawamura et al., 2002, 2003; Wu et al., 2003; Matsushita and Puro, 2006), on cerebral pericytes (Peppiatt et al., 2006; Fernández-Klett et al., 2010; Hill et al., 2015; Rungta et al., 2018), or both (Gonzales et al., 2020; Kovacs-Oller et al., 2020) have thus informed our current understanding of blood flow control and pericyte physiology. Although it is clear that a high degree of similarity exists between these vascular beds, the possibility of yet-to-be-identified differences between these networks should be borne in mind when attempting to draw generalizations from data from both vascular beds. To this end, we note explicitly where data on pericytes in this review were drawn from studies performed in retina.

**Figure 6:**
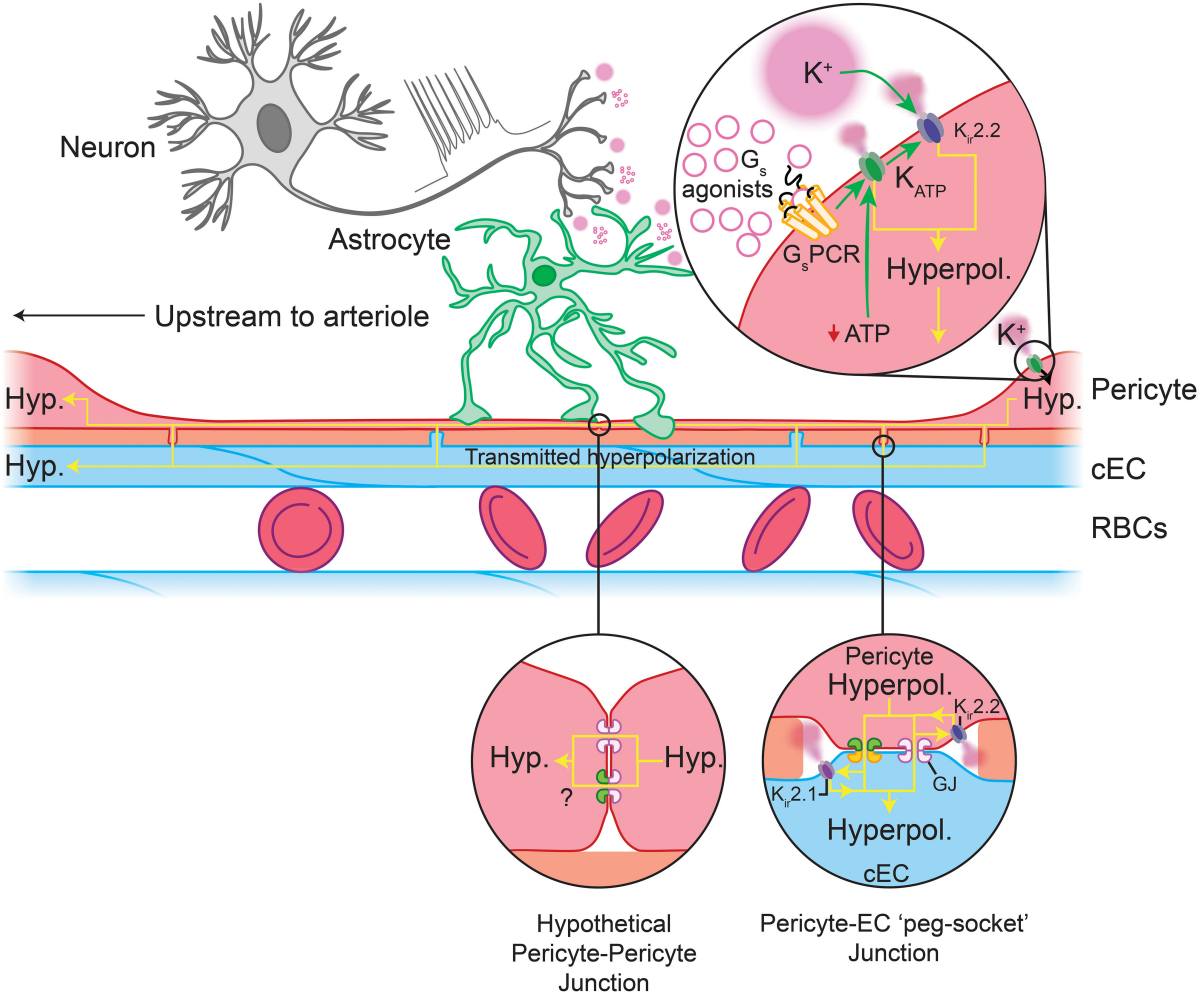
Predicted capillary pericyte-EC interactions to control local blood flow. Neuronal activity drives the release of K^+^ and G_s_PCR agonists. *Top inset:* These are predicted to engage pericyte K_ir_2.2 and their cognate GPCRs, respectively. G_s_PCR activity activates K_ATP_ channels, the hyperpolarization by which may feed forward to evoke further K_ir_2.2 activity (a sufficient fall in ATP:ADP would also engage K_ATP_ channels). The hyperpolarization generated by these channels may then be passed via gap junctions to cECs (*bottom right inset*) or possibly to adjacent pericytes, though direct pericyte-pericyte gap junctions have not been observed to date. In cECs, the incoming hyperpolarization will engage K_ir_2.1 channels to amplify hyperpolarization to a sufficient level to pass to adjacent cECs and pericytes. Hyperpolarization-mediated activation of K_ir_2.1 and K_ir_2.2 in these cells will rapidly regenerate the current so that it can be passed to the next cell, and so on upstream to the arteriole. Upon arrival at the arteriole and its first few offshoots, hyperpolarization will be passed via GJs at MEPs to SMCs and to contractile mural cells, which will close VDCCs, leading to a fall in intracellular Ca^2+^, relaxation of their actin-myosin contractile machinery, vasodilation, and an increase in blood flow.

What would be the physiological consequence of such profound membrane hyperpolarization in pericytes? It has been proposed that K_ATP_-generated hyperpolarization of pericytes in the retinal vasculature could be transmitted over long distances to close VDCCs in the mural cells of upstream vessels, thereby causing vasorelaxation and an increase in blood flow (Ishizaki et al., 2009). Such a mechanism could be enabled by transmission of hyperpolarizing signals either between pericytes themselves, or between pericytes and ECs. Indeed, hyperpolarizations transmitted to cECs are predicted to engage K_ir_2.1 channels, which we have recently shown to rapidly propagate hyperpolarizing signals over long distances through the brain endothelium to upstream arterioles, causing their dilation and an increase in blood flow (Longden and Nelson, 2015; Longden et al., 2017). A similar mechanism involving both K_ATP_ and K_ir_2.1 channels has also recently been shown to be critical for control of blood flow in the heart (Zhao et al., 2020). In the brain, connexin (Cx)37, and Cx45 are highly expressed in pericytes (He et al., 2018; Vanlandewijck et al., 2018; see Box 1), and thus these likely form cell-cell GJs that facilitate long-range transmission of K_ATP_-mediated electrical signals (Figure 6).

K_ir_2 channels are activated not only by membrane hyperpolarization, but also by external K^+^, which is an important mediator of NVC (Filosa et al., 2006; Longden and Nelson, 2015; Longden et al., 2017). Neurons or astrocytes release K^+^ into the perivascular space during NVC, and its concentration can reach ~10 mM during concerted activity (Orkand et al., 1966; Newman, 1986; Ballanyi et al., 1996; Kofuji and Newman, 2004). Interestingly, K_ir_2.2 channels are expressed in pericytes (Table 1 and Figure 5B) and K_ir_ currents with the expected biophysical characteristics and sensitivity to micromolar barium (Ba^2+^) have been reported in cultured retinal and heart pericytes (von Beckerath et al., 2000; Quignard et al., 2003), and retinal and kidney pericytes from microvessels (Cao et al., 2006; Matsushita and Puro, 2006). Strong rectification in K_ir_2 channels results from intracellular polyamine and Mg^2+^ block of the channel pore at depolarized membrane potentials, limiting outward current. This block is relieved by elevating external K^+^ to levels that are typically seen during neuronal activity, initiating rapid and self-perpetuating hyperpolarization that drives V_m_ toward E_K_ (Longden and Nelson, 2015). Thus, pericyte K_ir_2.2 channels could contribute to transmitted hyperpolarizations in several ways. On one hand, K^+^ elevations resulting from neural activity may directly activate K_ir_2.2 channels on pericytes (Figure 6). Alternatively, engagement of pericyte K_ATP_ channels could cause a K^+^ or hyperpolarization-mediated recruitment of K_ir_2.2 channels, which would serve to amplify hyperpolarization. K_ir_2.2 channels could then propagate hyperpolarizing signals from capillary pericytes to upstream vessels by means of pericyte-pericyte communication through their thin-strand processes or by passing hyperpolarization to neighboring ECs via pericyte-endothelial GJs. PIP_2_ is also central to K_ir_2 channel function (D'Avanzo et al., 2010; Hansen et al., 2011), and its depletion via G_q_PCR signaling has recently been shown to play an important role in regulating K_ir_2.1 channel activity in cECs (Harraz et al., 2018). Accordingly, signaling processes that influence PIP_2_ levels are anticipated to factor in to K_ir_2.2 channel activity in pericytes.

Collectively, genetic and functional data to date argue for an important role of K_ATP_ and K_ir_2.2 channels in regulating pericyte electrical activity, and we thus propose that the activity of these channels plays a central role in the control of capillary blood flow (Figure 6).

### Voltage-Gated K^+^ (K_v_) Channels Provide Graded Opposition to Membrane Depolarization

K_v_ channels are formed by 4 identical subunits that surround a central pore. Each subunit is composed of six transmembrane segments (S1–S6) of which four form the voltage sensor domain (S1–S4) with several regularly spaced positively-charged amino acids in the S4 helix playing a central role in transducing voltage into conformational changes that gate the channel. The remaining two transmembrane regions line the K^+^-selective pore (S5–S6; Figure 5C; Jiang et al., 2003; Chen et al., 2010).

In order of mRNA abundance, cerebral pericytes express modest to low levels of genes encoding: K_v_6.1, K_v_7.4, K_v_2.1, K_v_9.3, K_v_9.1, K_v_7.5, and K_v_1.2, in the absence of K_v_ beta subunits (Table 1). Outward K^+^ currents attributable to K_v_ channels have been measured in these cells, for example in guinea pig cochlear stria vascularis and cultured bovine retinal pericytes (von Beckerath et al., 2000; Quignard et al., 2003; Liu et al., 2018). K_v_ channels are crucial for negative feedback regulation of V_m_, their P_o_ and unitary currents increasing with membrane depolarization to provide a counterbalancing hyperpolarizing influence (Nelson and Quayle, 1995; Koide et al., 2018). Their activity can also be modulated by a range of intracellular signaling cascades that engage varied effectors such as PKC, c-SRC or Rho-kinase (which inhibit K_v_ channels) or cAMP-PKA and cyclic guanosine monophosphate(cGMP)-protein kinase G (PKG) signaling pathways (which promote channel activity) (Jackson, 2018). Of note, nitric oxide (NO) can exert major signaling effects via soluble guanylate cyclase (sGC) and cGMP-PKG in pericytes (Denninger and Marletta, 1999). As adjacent cECs are a major source of local NO (Longden et al., 2019), its elevation may be sufficient to engage pericyte PKG signaling to promote activity of K_V_ and other PKG-sensitive channels.

Cerebral arteriolar SMCs are each estimated to express ~3,000 K_v_ channels/cell (Dabertrand et al., 2015) composed principally of K_v_1.2 and K_v_1.5 (Straub et al., 2009) with activation initially detectable above −40 mV and increasing e-fold per 11-13 mV, exhibiting half-activation between approximately −10 and 0 mV (Robertson and Nelson, 1994; Straub et al., 2009). These channels also exhibit substantial steady-state inactivation over the physiological voltage range (Robertson and Nelson, 1994). K_v_ currents with similar characteristics have been described in cultured retinal pericytes (Quignard et al., 2003), whereas the half-maximal activation of K_v_ channels recorded in cultured coronary pericytes is substantially more negative at −40.9 mV, along with a steeper voltage-dependence of activation (e-fold per 4.6 mV) and only modest inactivation at physiological membrane potentials (von Beckerath et al., 2000). Thus, K_v_ current characteristics in pericytes appear to be regionally dependent, likely a result of differential expression and assembly of distinct K_v_ isoforms. Direct characterization of K_v_ currents in native brain pericytes is therefore critical to furthering our understanding of their role in the control of pericyte V_m_, where these channels are anticipated to provide negative feedback to limit depolarization effected by the activity of depolarizing ion channels in pericytes, such as those of the TRP family.

### K_2P_3.1 Channels Provide a Background K^+^ Conductance and May Impart pH Sensitivity

K_2P_ channels contribute to maintenance of resting membrane potential due to steady outward K^+^ “leak” at potentials positive to E_K_. They comprise a family of 15 members, and are composed of two identical subunits, each with four transmembrane domains with two pore-forming loops making up a central K^+^-conducting pore (Figure 5D; Miller and Long, 2012; Lolicato et al., 2014). K_2P_3.1, also known as the two-pore domain weakly inwardly-rectifying K^+^ channel (*T*WIK)-related *a*cid-*s*ensitive *K*^+^ (TASK)-1 channel (Duprat et al., 1997), is the only K_2P_ isoform expressed in capillary pericytes, and is also expressed in cerebral SMCs (He et al., 2018; Vanlandewijck et al., 2018). In SMCs, its steady current contributes to maintaining a relatively negative V_m_ by counterbalancing depolarizing influences (Gurney et al., 2003).

Perhaps the most well-studied characteristic of TASK-1 is its sensitivity to pH within the range of ~6.5–8. Acidic pH inhibits channel activity while alkaline pH increases it, with half-maximal activation occurring at pH 7.4 and ~90% of maximal TASK-1 current recorded at pH 7.7 (Duprat et al., 1997). Synchronous neuronal activity can cause rapid changes in pH. For example, alkalization in extracellular pH has been observed in the hippocampus, cerebellum and some cortical areas, by up to 0.2 units (Chesler and Kaila, 1992; Makani and Chesler, 2010). Thus, it is possible that in addition to setting resting V_m_, K_2P_3.1 imparts sensitivity to pericytes in these regions to such shifts, which could hyperpolarize V_m_ to modulate blood flow through the mechanisms described above.

### Na^+^- and Ca^2+^-Activated K^+^ Channels Are Expressed at Low Levels in Pericytes

Capillary pericytes also express low levels of genes encoding the Na^+^-activated K_Na_1.2 channel and the Ca^2+^-activated K_Ca_2.3 channel (Table 1). K_Na_1.2 channels (Figure 5E) are sensitive to intracellular Na^+^ and Cl^−^, and are dramatically stimulated by cell swelling and inhibited by a decrease in cell volume (Bhattacharjee et al., 2003; Tejada et al., 2014). Thus, they could impart sensitivity to pericyte volume changes, and may respond to fluctuations in intracellular ion concentrations or metabolic state.

K_Ca_2.3 (also known as SK3) belongs to the family of small-conductance Ca^2+^-activated K^+^ (SK) channels that share overall transmembrane topology with K_v_ channels, yet lack a functional voltage-sensor at S4 (Figure 5F; Adelman et al., 2012). Each subunit in the tetrameric channel is associated with a calmodulin (CaM) monomer via a CaM binding domain in the C-terminal region. Ca^2+^ binding to CaM induces a conformational change which leads to rapid channel opening, with an EC_50_ for Ca^2+^ of 300–500 nM (Ledoux et al., 2006; Adelman et al., 2012). If functional SK channels in native pericytes are confirmed, they are expected to facilitate coupling between Ca^2+^ elevations and membrane hyperpolarization.

## Pericyte TRP Channels

**Figure 7:**
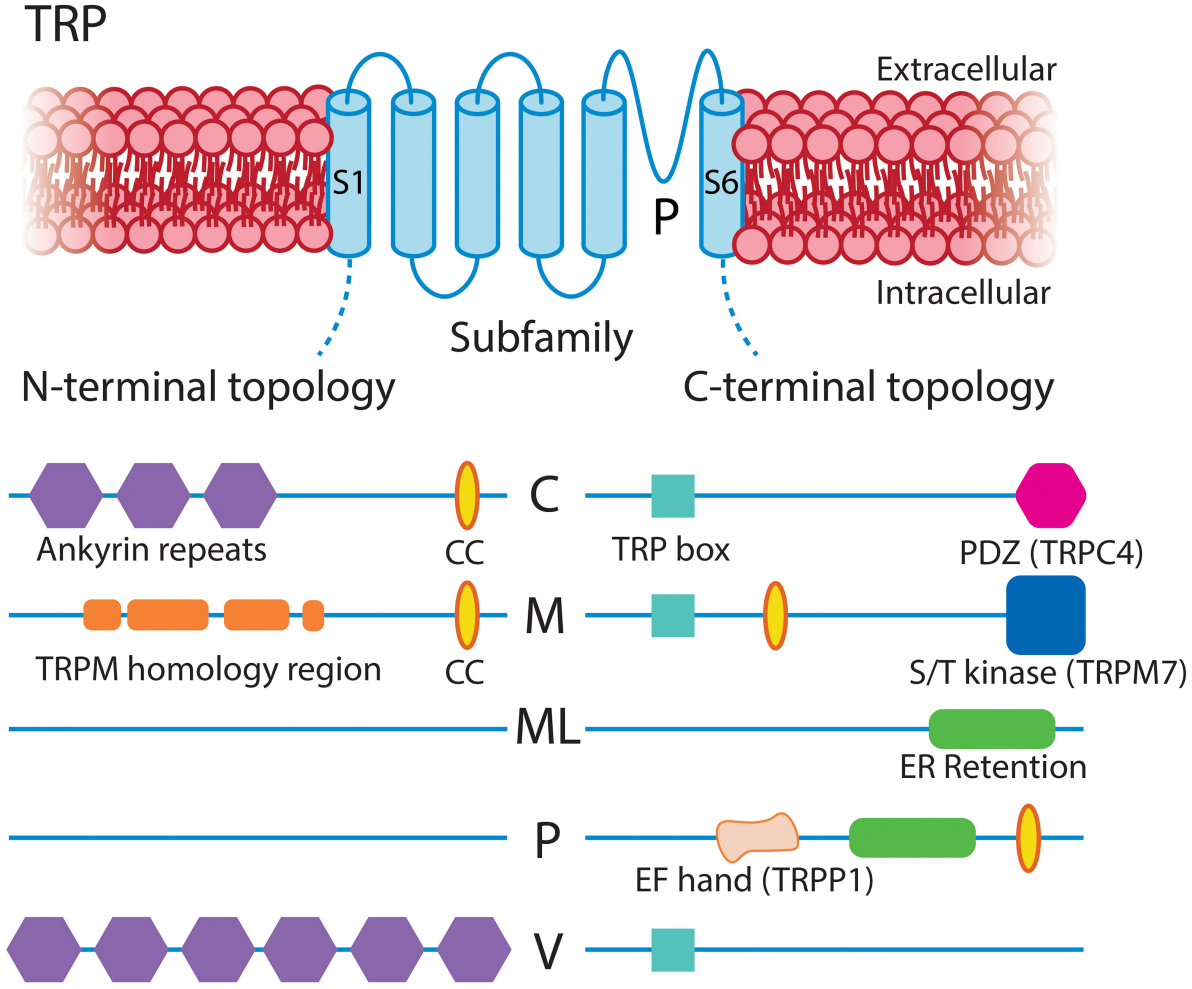
Structural overview of the TRP families expressed in CNS capillary pericytes, adapted with permission from Clapham (2003). All TRP channels share a common and typical 6-transmembrane structure with profoundly varying intracellular N- and C-terminal domains, the major features of which are illustrated. CC, coiled-coil domain.

The TRP channel family mediates cellular responses to a wide range of stimuli (Clapham, 2003). These are non-selective cation channels that depolarize the membrane upon activation and, in many cases, conduct significant amounts of Ca^2+^. In mammals there are six subfamilies of TRP channels encoded by 28 genes, 11 of which are expressed by capillary pericytes. These are canonical (TRPC1, TRPC3, TRPC4, TRPC6), melastatin (TRPM3, TRPM4, TRPM7), mucolipin (TRPML1), poly-cystin (TRPP1, TRPP3), and vanilloid (TRPV2) channels (Earley and Brayden, 2010; He et al., 2018; Vanlandewijck et al., 2018). Functional TRP channels are tetramers of subunits with a common six transmembrane structure, which can assemble into homomeric or heteromeric functional channels. Their tendency to heteromerize, generally with closely related members, can give rise to channels with unique sensing capabilities and biophysical properties (Venkatachalam and Montell, 2007). Overall, subfamily members share ~35% amino acid sequence homology, with the majority of this diversity arising from differences in their cytoplasmic domains (Figure 7; Clapham, 2003; Nilius and Owsianik, 2011). While they have been traditionally described as “non-selective,” the pattern of ion selectivity for different cations varies between subfamilies (Hill-Eubanks et al., 2014; see Table 1).

Broadly speaking, TRP channels are major downstream effectors for GPCR signaling (Clapham, 2003; Veldhuis et al., 2015), with particular second messenger systems both activating or sensitizing some TRP channels, and decreasing the activity of others. TRPC channels are Ca^2+^ permeable and typically activated by plasmalemmal GPCRs or tyrosine kinase receptors that activate PLC isoforms (Albert, 2011). TRPC3/6 channels are directly activated by DAG, which is liberated by G_q_ signaling, and inhibited by PIP_2_, which decreases during G_q_ activity (Hofmann et al., 1999; Albert, 2011). The activation mechanisms of TRPC4 are less clear, whereas TRPC1-containing channels are unresponsive to DAG and are instead gated by PIP_2_ in a PKC-dependent manner (Hofmann et al., 1999; Albert, 2011), although heteromultimerization with TRPC3 can convey DAG sensitivity (Lintschinger et al., 2000). TRPC3 is the most robustly expressed TRP channel in capillary pericytes (Table 1) and is thus likely to be engaged during G_q_PCR-DAG signaling. This channel permits robust Ca^2+^ entry, although it has relatively low selectivity for Ca^2+^ over Na^+^ (*p*Ca^2+^:*p*Na^+^ ~1.5; Pedersen et al., 2005). At the arteriolar level, TRPC3 has been implicated in mediating vasodilation through elevations of EC Ca^2+^ leading to K_Ca_2.3 activation (Kochukov et al., 2014), whereas its activation in SMCs mediates arteriolar constriction through a mechanism involving an IP_3_R-activated (sarcoplasmic reticulum (SR) Ca^2+^ release independent) TRPC3-dependent Na^+^ current that depolarizes V_m_ and activates VDCCs (Xi et al., 2009). Similar couplings may occur in capillary pericytes, likely depending on the macromolecular organization of TRPC3 with other local signaling elements.

Members of the TRPC subfamily, in particular TRPC1, have also been suggested to participate in store-operated Ca^2+^ entry (SOCE)—an event activated by the depletion of endoplasmic reticulum (ER) Ca^2+^ stores that depends on Orai1 and the ER-Ca^2+^ status sensing protein stromal interaction molecule 1 (STIM1; Huang et al., 2006; Soboloff et al., 2006; Cheng et al., 2008, 2013). Capillary pericytes express STIM1 and Orai1 and 3 (Table 1), and thus a functional interaction between TRPC1 and these proteins could be important for SOCE in pericytes. Recent work also shows TRPM7 activation, although not essential, can positively modulate SOCE (Souza Bomfim et al., 2020).

The melastatin channel TRPM4 is unique in its exclusive permeability to monovalent cations. Na^+^ currents through TRPM4 are voltage-dependent and activated by intracellular Ca^2+^ (EC_50_ ~20 μM) with the Ca^2+^ sensitivity of the channel regulated by multiple factors including cytosolic ATP, PKC-dependent phosphorylation and calmodulin (Nilius et al., 2005; Ullrich et al., 2005). In cerebral SMCs, membrane stretch indirectly activates TRPM4 (and TRPC6) current through angiotensin II AT_1_ receptor activation and a resultant IP_3_-mediated Ca^2+^ elevation (Gonzales et al., 2014). Pericytes also express the AT_1_ receptor, and thus a similar mechanism may be present in capillary pericytes which could contribute to the mild, slow constrictions these cells are capable of Fernández-Klett et al. (2010). In contrast to the monovalent conductance of TRPM4, the closely related TRPM3 and TRPM7 channels are also permeable to Ca^2+^ and Mg^2+^ (Pedersen et al., 2005). TRPM3 is activated by cell swelling, the neurosteroid pregnenolone sulfate, and the metabolite D-erythro-sphingosine and related sphingosine analogs and thus may impart sensitivity to steroid and lipid signals to pericytes (Grimm et al., 2005; Wagner et al., 2008). As pericytes also robustly express the S1P_3_ receptor (discussed below), it is likely that TRPM3 and S1P_3_ respond in concert to locally released lipids, such as those released constitutively by ECs and RBCs (Selim et al., 2011; Ksiazek et al., 2015). TRPM7, in contrast, is ubiquitously expressed and plays a major role in Mg^2+^ homeostasis (Schlingmann et al., 2007).

Functional TRPP1 channels (encoded by the *Pkd2* gene) have a large conductance and conduct a significant amount of Ca^2+^ (Earley and Brayden, 2015). This channel has been implicated in mechanosensation when expressed alongside polycystic kidney disease (PKD)1 (Giamarchi and Delmas, 2007; Sharif-Naeini et al., 2009; Narayanan et al., 2013). As PKD1 is also present in pericytes, these channels may aid in the detection of local mechanical forces, such as paravascular fluid shear from the glymphatic system (Mestre et al., 2018), or those imparted through the very thin endothelium by changes in blood flow during neuronal activity, or through subtle changes in diameter of the underlying capillary. Similarly, the vanilloid family member TRPV2, also expressed in SMCs throughout the vasculature (Muraki et al., 2003), has been suggested to play a role in mechanosensation-evoked Ca^2+^ entry (Perálvarez-Marín et al., 2013). Continuing this theme, mechanosensory contributions have also been reported for TRPC1, TRPC6, and TRPM4 (Yin and Kuebler, 2010). Combined with the fact that pericytes also express Piezo1 (see below), this represents a broad mechanosensing repertoire, suggesting that pericytes may be exquisitely sensitive to a range of mechanical perturbations. The resultant Ca^2+^ elevation and depolarizing currents through the activity of these channels could couple to a number of processes, including driving further Ca^2+^ release from stores, and activation of VDCCs, K_Ca_2.3 channels, or Ca^2+^-activated Cl^−^ channels (CaCCs; discussed below). As recent work demonstrates that pericytes can subtly influence tone throughout the capillary bed (Fernández-Klett et al., 2010), mechanosensing and Ca^2+^-mediated mechanisms may play an important role in influencing this process.

## Pericyte Ca^2+^ Channels

The overall expression level of Ca^2+^ channels is similar to that of TRP channels in pericytes, composed of message for IP_3_R subtypes and a range of VDCCs.

### IP_3_Rs Permit a Versatile Range of Ca^2+^ Signaling Behaviors in Response to Extracellular Signals

**Figure 8:**
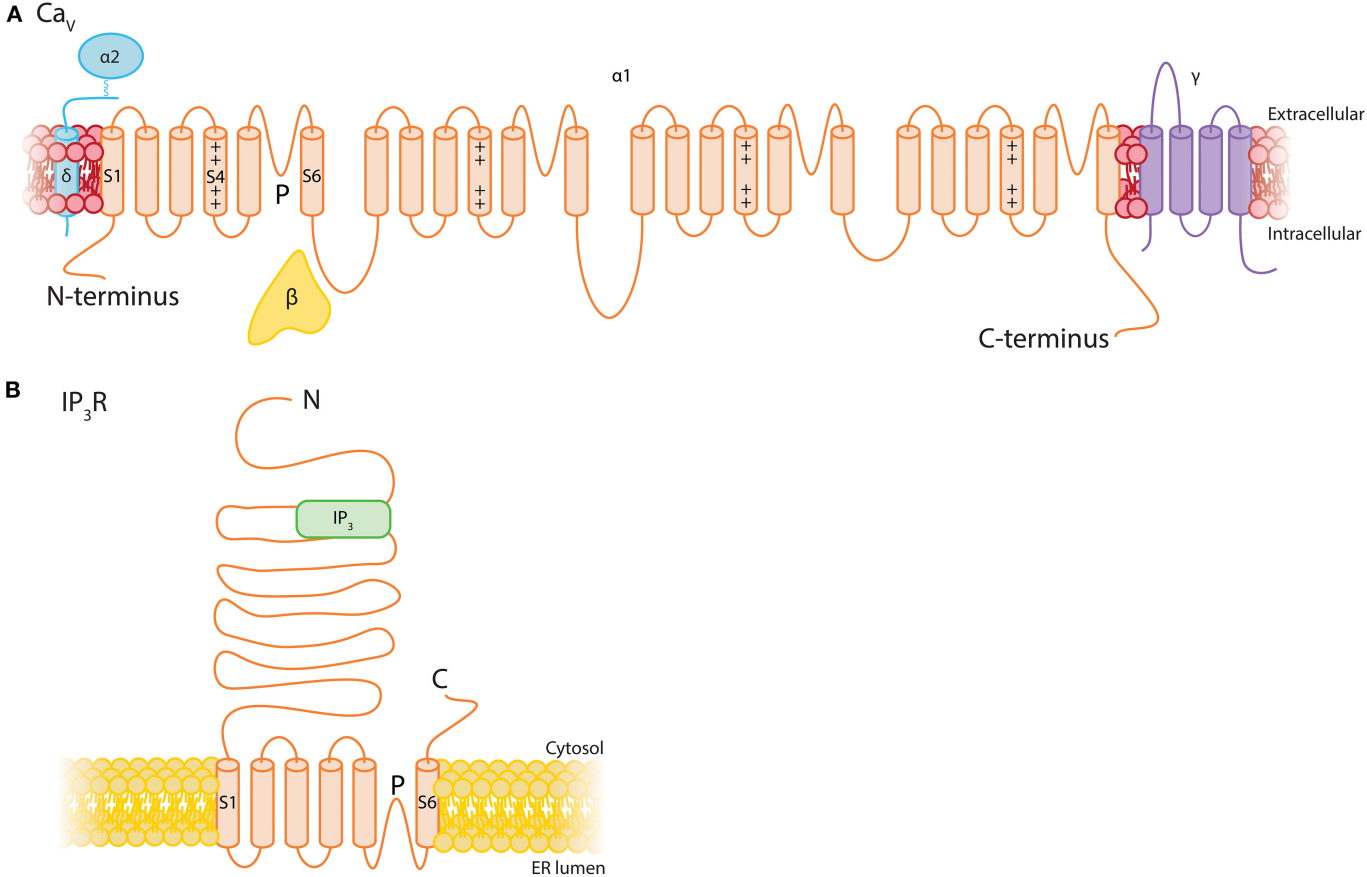
Structural topology of Ca^2+^ channels expressed by pericytes. **(A)** The general structure of Ca_v_ channels consists of a single 24-transmembrane α subunit which is a repeat of a 6-transmembrane motif with an embedded voltage sensor connected by intracellular loops. This is accompanied by associated β, γ, and α2δ subunits. **(B)** IP_3_Rs consist of a tetrameric assembly of 6- transmembrane subunits with a large N-terminal domain that contains the IP_3_ binding site.

The vast majority of intracellular Ca^2+^ signals arise from either Ca^2+^ influx across the plasmalemma, or release from the SR/ER via IP_3_Rs or ryanodine receptors (RyRs). IP_3_Rs are enormous proteins (~1.3 MDa) formed by four IP_3_R subunits. Three subunit isoforms—IP_3_R1-3—exist, which are able to homo- or heterotetramize. Each individual subunit has six transmembrane segments: The fifth and sixth segments form a central ion-conducting pore that is connected via a linker to the peripheral bundle formed by transmembrane domains 1-4. The large cytoplasmic N-terminal domain contains the IP_3_ binding site and a putative Ca^2+^ sensor region, and binding of IP_3_ and Ca^2+^ leads to conformational changes which are transmitted to the pore to gate the channel (Figure 8; Fan et al., 2015; Baker et al., 2017; Hamada et al., 2017). IP_3_R subtypes share ~70% homology and differ in their affinity for IP_3_, with IP_3_R2 being more sensitive than IP_3_R1, and both of these subtypes being more sensitive than IP_3_R3 (Tu et al., 2005; Iwai et al., 2007). Brain capillary pericytes express the genes encoding IP_3_Rs 1 and 2 robustly, and a much lower level of IP_3_R3, whereas RyRs are not appreciably expressed by these cells (He et al., 2018; Vanlandewijck et al., 2018; Table 1).

As described briefly above, G_q_PCRs activating phospholipase Cβ (PLCβ) (Fisher et al., 2020), or receptor tyrosine kinases (RTKs) activating PLCγ, can mediate the formation of IP_3_ and DAG from PIP_2_. IP_3_ then binds to IP_3_Rs on the ER membrane, leading to Ca^2+^ release from the ER lumen (where Ca^2+^ is maintained between 100 and 800 μM; Burdakov et al., 2005) down its electrochemical gradient into the cytosol (<100 nM basal Ca^2+^; Berridge, 2016). IP_3_ and Ca^2+^ act as co-agonists at IP_3_Rs (Bezprozvanny et al., 1991; Finch et al., 1991; Foskett et al., 2007) and channels display a biphasic sensitivity to Ca^2+^, resulting in a characteristic bell-shaped concentration-response curve. In the presence of very low IP_3_ levels, IP_3_Rs are extremely sensitive to Ca^2+^ inhibition. However, a small increase in IP_3_ concentration (to ~100 nM) profoundly reduces the sensitivity of the channel to Ca^2+^ inhibition, permitting dramatic increases in activity (Iino, 1990; Bezprozvanny et al., 1991; Finch et al., 1991; Foskett et al., 2007).

The resultant release of stored Ca^2+^ can take on a broad range of spatiotemporal profiles, which depend on many factors. To name just a few, these include the concentration of local IP_3_ and Ca^2+^, ER Ca^2+^ load, the type, and number of IP_3_Rs expressed, their splice variation, whether they are homomers or heteromers, and the topology of the local microenvironment. Such intricacies provide the versatility to potentially generate a huge variety of Ca^2+^ signals that encode information through their amplitudes, durations, frequencies, and spatial characteristics (Bootman and Bultynck, 2020). Despite these inherent complexities, a range of stereotyped IP_3_R-mediated Ca^2+^ signals typically emerge. These range from the opening of single IP_3_R (termed a “blip”), to the coordinated, weakly cooperative openings of a cluster of around 6 IP_3_Rs within a release site (a “puff”), to finally—with sufficient IP_3_–a long-range regenerative Ca^2+^ “wave” arising due to the recruitment of successive sites through the process of Ca^2+^-induced Ca^2+^ release (CICR) (Berridge et al., 2000; Smith and Parker, 2009; Lock and Parker, 2020).

Store-mediated Ca^2+^ release has been observed in pericytes in a range of contexts. For example, pericytes of the ureter display long-duration IP_3_R-mediated Ca^2+^ transients in response to the G_q_PCR agonists endothelin-1 and arginine vasopressin. These signals are suppressed by elevations of Ca^2+^ in adjacent cECs, which are suggested to inhibit IP_3_R activity through a NO-dependent mechanism (Borysova et al., 2013). Spontaneous ER Ca^2+^ release-dependent Ca^2+^ transients have also been observed in suburothelial capillary pericytes, which activate CaCCs to depolarize the membrane, subsequently recruiting VDCCs (Hashitani et al., 2018).

In the brain, recent studies have revealed that capillary pericytes generate microdomain Ca^2+^ oscillations under ambient conditions, and that neural activity evoked by odor leads to a transient cessation of these signals and a decrease in basal Ca^2+^, which correlates with an increase in RBC velocity (Hill et al., 2015; Rungta et al., 2018). However, it is worthy of note that a decrease was not observed in similar experiments in which whisker stimulation was used to drive activity (Hill et al., 2015), suggesting the possibility of heterogeneity in the Ca^2+^ signaling machinery deployed by pericytes in different regions of the cortex. The specific ion channels and broader mechanisms that underlie these ambient signals have not yet been delineated, but IP_3_Rs are obvious potential candidates. Elucidation of the mechanistic basis and roles of these Ca^2+^ signals in brain capillaries is critical, and awaits further experimentation.

### Voltage-Dependent Ca^2+^ Channels Directly Link V_m_ to Ca^2+^ Entry

VDCCs are composed of four to five distinct subunits (α_1_, β, α_2_δ, and γ; Figure 7). The α_1_ subunits are pore forming and responsible for the pharmacological diversity of different VDCC subtypes. These are associated with an intracellular β subunit, a disulphide-linked α_2_δ subunit, and in some cases a transmembrane γ subunit, each of which regulate surface expression and tune the biophysical properties of the channel (Catterall et al., 2005). The large α_1_ subunit is organized into four homologous domains, each comprising six transmembrane segments (S1-S6) with intracellular N- and C- termini. Similar to K_v_ channels, the S4 segment of each of these domains comprises the voltage sensor and the S5-S6 regions form the ion conducting pore (Catterall et al., 2005). Capillary pericytes express genes encoding the α subunits for L-type (Ca_v_1.2, Ca_v_1.3), P/Q-type (Ca_v_2.1), and T-type (Ca_v_3.1, Ca_v_3.2) channels and thus we briefly review the salient properties of these here. They also express low levels of several genes encoding β and α_2_δ auxiliary subunits (He et al., 2018; Vanlandewijck et al., 2018).

As with K_v_ channels, VDCC activity depends on membrane potential: P_o_ steeply increases with depolarization, balanced by multiple feedback mechanisms that act to limit Ca^2+^ entry at depolarized potentials. Prominent among these are voltage- and Ca^2+^-dependent inactivation. Voltage-dependent inactivation (VDI) is inherent to the α_1_ subunit but is modulated by the ancillary β subunit and others, whereas Ca^2+^-dependent inactivation (CDI) is conferred by a CaM monomer associated with the α_1_ carboxy tail (Peterson et al., 1999; An and Zamponi, 2005; Dick et al., 2008; Tadross and Yue, 2010; Tadross et al., 2010). Regulation is additionally complicated by the panoply of alternative splice variants that can be expressed, which impact the biophysical properties of the functional channel, including sensitivity to CDI and VDI.

L-type channels are widely expressed, including in the heart, in skeletal and smooth muscle, and in neurons (Zamponi et al., 2015). Ca_v_1.2 and Ca_v_1.3 have distinct biophysical and pharmacological differences (Lipscombe et al., 2004)—Ca_v_1.3 channels open and close on faster timescales than Ca_v_1.2 (Helton et al., 2005), and are less sensitive to inhibition by dihydropyridines (Xu and Lipscombe, 2001). A C-terminal modulatory (CTM) domain can structurally interfere with CaM binding to decrease P_o_ and reduce CDI, an effect that is more pronounced in Ca_v_1.3 than Ca_v_1.2 (Striessnig et al., 2014). Moreover, in alternatively spliced Ca_v_1.3 channels, the absence of a CTM domain can shift the voltage of half-maximal activation by ~+10 mV by decreasing the slope factor of the activation curve without any effects on activation threshold (Singh et al., 2008). At physiological extracellular Ca^2+^ levels, the activation threshold of Ca_v_1.3 is much more negative (-55 mV) than Ca_v_1.2 (-25 to −30 mV) (Xu and Lipscombe, 2001). Thus, at pericyte resting V_m_ of around −45 mV, as measured in the retina (Zhang et al., 2011), Ca_v_1.3 channels could be active and contribute to Ca^2+^ entry.

In addition to voltage- and Ca^2+^-dependent inhibition, L-type VDCC activity is heavily regulated by GPCR signaling. Prominent among these, G_s_-cAMP-PKA signaling has long been known to play an important role in stimulating channel activity, and has been studied extensively in the heart. Here, it was recently shown that the target of PKA phosphorylation is not the core channel itself, as mutation of all PKA consensus phosphorylation sites to alanine resulted in channels that retained PKA regulation. Rather, PKA acts via the small G protein Rad, a constitutive inhibitor of VDCCs. Phosphorylation of Rad relieves its interaction with β subunits, and allows channel activity (Liu et al., 2020). Further regulation of L-type channels by PKC, stimulated by DAG liberated as a result of G_q_PCR activity, is also a possibility, with both inhibitory and potentiating effects having been observed (Kamp and Hell, 2000).

P- and Q-type currents are both attributable to Ca_v_2.1, with the β subunit accompanying the pore-forming subunit thought to account for their differences (Zamponi et al., 2015). These channels have been best characterized in the nerve terminals and dendrites of neurons where they couple Ca^2+^ entry with neurotransmitter release (Zamponi et al., 2015) and also play a role in coupling Ca^2+^ entry to gene transcription via engagement of CaM kinase II (Wheeler et al., 2012). They open in response to similar depolarization levels as Ca_v_1.2 channels, with an activation threshold of approximately −40 mV (Adams et al., 2009). Upon repetitive/tetanic stimulation, as occurs during neuronal activity, CaM can bind to two adjacent sites on the Ca_v_2.1 α_1_ subunit to mediate an initial Ca^2+^-dependent facilitation (CDF) of P/Q-type current, followed by progressive CDI, with a relatively slow (30 s−1 min) recovery from this (Lee et al., 1999, 2000). While CDI of Ca_v_2.1 requires a global Ca^2+^ increase, CDF can be promoted by Ca^2+^ entry through an individual Ca_v_2.1 channel and results in an enhancement of channel P_o_, enabling stimulation-evoked increases in amplitude and duration of Ca^2+^ currents (Chaudhuri et al., 2007). Slow and fast modes of Ca_v_2.1 gating have been proposed. The slow mode exhibits longer mean closed times and latency to first opening, slower kinetics of inactivation, and necessitates larger depolarizations to open the channel. Inactivation also occurs at more depolarized potentials in the slow compared to fast mode (Luvisetto et al., 2004). The type of β subunit modulates the prevalence of these modes, with fast and slow gating mediated by β_3a_ and β_4a_ subunits, respectively (Luvisetto et al., 2004), the latter of which is expressed more robustly by brain pericytes (He et al., 2018; Vanlandewijck et al., 2018). Ca_v_2.1 channels are inhibited by GPCR activity through several distinct mechanisms—direct binding of the G protein βγ dimer can augment VDI, while voltage-independent mechanisms such as phosphorylation, depletion of essential lipids, and trafficking mechanisms also play important roles (Zamponi and Currie, 2013).

T-type (Ca_v_3.1 and Ca_v_3.2) channels are activated at more negative potentials, around −60 mV, with rapid gating kinetics and small single channel amplitudes (Iftinca and Zamponi, 2009; Rossier, 2016). At membrane potentials of −65 to −55 mV, these channels exhibit window currents in which the channels open but do not inactivate completely, permitting ongoing Ca^2+^ entry (Perez-Reyes, 2003). These channels can be modulated by the activity of a broad range of GPCRs, including those with Gα subunits that couple to PKA, PKC, and PKG, along with direct effects of Gβγ subunits (Iftinca and Zamponi, 2009).

Both L- and T-type VDCCs are expressed in cerebral SMCs (Hill-Eubanks et al., 2011; Harraz and Welsh, 2013; Harraz et al., 2014). Here, L-type channels provide Ca^2+^ for contraction (Nelson et al., 1990), whereas T-type channels provide negative feedback by coupling Ca^2+^ entry to RyR activity. Subsequent Ca^2+^ release via RyRs in turn activates large-conductance Ca^2+^-activated K^+^ (BK) channels to hyperpolarize the membrane (Harraz and Welsh, 2013; Harraz et al., 2014). T- and P/Q-type channel currents have not yet been observed in native pericytes, but L-type VDCC currents have been measured in the retina (Sakagami et al., 1999). Variance in the magnitude of L-type VDCC Ca^2+^ currents across the microvascular network has functional consequences for the degree of Ca^2+^ entry via these channels (Matsushita et al., 2010; Burdyga and Borysova, 2014). In the retina, L-type VDCC currents are 7.5-fold higher in SMCs as compared to capillary pericytes, suggesting that V_m_ changes influence intracellular Ca^2+^ levels to a greater degree at the level of arterioles (Matsushita et al., 2010). Indeed, extracellular K^+^ at 10 mM (a concentration that evokes K_ir_-mediated hyperpolarization) and 97.5 mM (which depolarizes the membrane to drive VDCC activity) significantly decreased and increased intracellular Ca^2+^ in arteriolar SMCs, respectively, but had only a marginal effect on capillary pericyte Ca^2+^ (Matsushita et al., 2010). Thorough characterization of native brain capillary pericyte VDCC currents and their densities is needed to advance our understanding of the contribution of these channels to pericyte Ca^2+^ handling.

## Pericyte Cl^−^ Channels

Cl^−^ channels are found in the plasma membrane and that of intracellular organelles and have been implicated in the regulation of cell excitability and volume, acidification of intracellular organelles, control of muscle tone, and synaptic transmission (Jentsch et al., 1999; Nilius and Droogmans, 2003). While they are permeable to other anions (such as iodide, bromide, or nitrate), they are referred to as Cl^−^ channels since this is the most abundant permeating anion species (Jentsch et al., 2002). Capillary pericytes express the CaCC formerly known as TMEM16A or anoctamin (Ano)1, and several members of the voltage-dependent chloride channel (ClC) family—ClC-2,−3,−4,−6, and−7 (He et al., 2018; Vanlandewijck et al., 2018). The latter four of these are Cl^−^/H^+^ antiporters and are not considered further here. Capillary pericytes also express other anoctamins that have been implicated in lipid scrambling: Ano4 and Ano6, as well as the poorly understood Ano10 (He et al., 2018; Vanlandewijck et al., 2018). Reports indicate that Ano6 may act as a Ca^2+^-activated Cl^−^ and non-selective cation channel with scramblase activity (Suzuki et al., 2010; Yang et al., 2012; Grubb et al., 2013) and Ano4 was recently shown to be a Ca^2+^-dependent non-specific cation channel with similar scrambling capabilities (Reichhart et al., 2019).

### CaCC Channels Couple Intracellular Ca^2+^ Increases to Depolarizing Cl^−^ Efflux

The CaCC TMEM16A is a homodimer of two pores and ten transmembrane domains, cytosolic N- and C-termini, and an extracellular domain (Dang et al., 2017; Paulino et al., 2017). Ca^2+^ binding to a transmembrane region of the pore induces a conformational rearrangement that gates the channel and leads to Cl^−^ permeation, generating a current that is outwardly rectifying with a slope conductance of ~8 pS (Yang et al., 2008; Xiao et al., 2011; Paulino et al., 2017). Ca^2+^ and voltage gating are closely coupled, with a stretch of 8 amino acids controlling both Ca^2+^ sensitivity and voltage-dependence of the channel (Xiao et al., 2011). Indeed, a remarkable feature of this channel is the voltage-dependence of Ca^2+^ sensitivity, with an EC_50_ of 2.6 μM at −60 mV and 400 nM at +60 mV. At physiological voltages, the channel is maximally activated by around 10 μM intracellular Ca^2+^ but concentrations exceeding this lower activation. Strong depolarization (above ~100 mV), in contrast, opens the channel even in the absence of Ca^2+^, despite the lack of a classic voltage sensor in the CaCC structure (Yang et al., 2008; Xiao et al., 2011). The kinetics of activation are slow at positive potentials, but are sharpened by an elevation of Ca^2+^, and at negative potentials channels display deactivation (Nilius and Droogmans, 2003). This interplay between V_m_ and intracellular Ca^2+^ makes the CaCC an attractive candidate for regulation of V_m_ in response to elevations intracellular Ca^2+^.

Since CaCC is sensitive to micromolar-range intracellular Ca^2+^ at typical resting potentials, it seems plausible that it is stimulated by local Ca^2+^ elevations (as opposed to global increases) such as those occurring through nearby TRPs, VDCCs, Orai channels, or IP_3_Rs. In keeping with this notion, cerebral SMC CaCCs are activated by TRPC6-mediated Ca^2+^ entry which drives vasoconstriction (Wang et al., 2016). Coupling of IP_3_R activity to CaCCs has also been reported in response to purinergic receptor activation, wherein CaCC-containing membrane domains are closely localized with ER regions via a physical linkage between this protein and IP_3_R1, facilitating exclusive communication between the two and exposing the CaCC to high Ca^2+^ concentrations during its release from the ER (Jin et al., 2013; Cabrita et al., 2017).

Underscoring their important role in the vasculature, targeted disruption of CaCCs from contractile vascular SMCs, mural cells and pericytes lowers systemic blood pressure (Heinze et al., 2014), whereas conversely CaCC overexpression drives hypertension (Wang et al., 2015). In vascular SMCs, the driving force for depolarizing Cl^−^ currents comes from Cl^−^/HCO3^−^ exchange and Na^+^/K^+^/Cl^−^ cotransport which enable high intracellular Cl^−^ concentrations (30–50 mM; Owen, 1984; Chipperfield and Harper, 2000; Kitamura and Yamazaki, 2001). Capillary pericytes in the brain express mRNA for genes encoding two of the SLC4 family Cl^−^/HCO3^−^ exchangers (*Slc4a2, Slc4a3*) and the NKCC1 Na^+^/K^+^/Cl^−^ cotransporter (*Slc12a2*) (He et al., 2018; Vanlandewijck et al., 2018), which raise the potential for similarly high intracellular Cl^−^ concentrations. E_Cl_ with 30–50 mM intracellular Cl^−^ and 133 mM extracellular Cl^−^ (Longden et al., 2016) is between approximately −35 and −25 mV—more positive than resting V_m_ of pericytes (~-45 mV, as measured in the retina; Zhang et al., 2011), therefore under these conditions activation of CaCC would cause Cl^−^ efflux and membrane depolarization, as seen in SMCs (Kitamura and Yamazaki, 2001; Bulley and Jaggar, 2014). While direct evidence for CaCCs in cortical capillary pericytes is currently lacking, in bladder pericytes ER Ca^2+^ release activates CaCCs and the resulting depolarization propagates to upstream SMCs of pre-capillary arterioles via gap junctions, where they depolarize the membrane to activate L-type VDCCs (Hashitani et al., 2018). In the pericytes of descending vasa recta, angiotensin II causes cytoplasmic Ca^2+^ oscillations that activate CaCC channels and depolarize V_m_ to approximately −30 mV (Zhang et al., 2008; Lin et al., 2010). CaCC current and membrane depolarization have also been recorded in retinal pericytes, where CaCC activation depends on unidentified non-selective cation channels (Sakagami et al., 1999) and can be evoked by G_q_PCR stimulation with endothelin (Kawamura et al., 2002). Thus, CaCCs in brain pericytes are predicted to depolarize V_m_ by coupling to a number of potential Ca^2+^ sources, including IP_3_Rs and TRP channels.

### ClC Channels May Repolarize the Membrane Following Electrical Signaling

ClCs are double-barreled homodimeric channels with one ion conduction pore per monomer (Dutzler et al., 2002). Each subunit is made up of 18 α-helices which display an interesting internal anti-parallel architecture, and many of these helices are shortened and tilted which permits disparate parts of the polypeptide to come together to form the Cl^−^ selectivity filter of the pore (Dutzler et al., 2002). The C-terminus also contains two cystathione-β-synthase domains, which regulate gating by binding ATP and ADP to decelerate the kinetics of activation and deactivation (Estévez et al., 2004; Stölting et al., 2013). ClC-2 has a unitary conductance of 2-3 pS and displays strong inward rectification. A remarkable biophysical characteristic of this channel is its slow hyperpolarization-mediated activation at potentials negative to around−40 mV, giving rise to currents that are only very slowly inactivating (Nilius and Droogmans, 2003; Bi et al., 2014). In addition to its hyperpolarization activation, it is sensitive to changes in cell volume and extracellular pH and is also activated by PKA (Nilius and Droogmans, 2003; Bi et al., 2014). As we have suggested previously for hyperpolarizing electrical signals generated in cECs, ClC-2 is an attractive candidate for mediating membrane repolarization (Garcia and Longden, 2020), in that its slow activation kinetics would enable K_ir_-mediated electrical signals to be generated and sent upstream before ClC-2 mediated Cl^−^ current fully develops to repolarize the membrane. Accordingly, ClC-2 may fulfill a similar role in pericytes to initiate membrane repolarization in the wake of electrical signals generated by K_ATP_ and K_ir_ channels.

## Further Channels in Pericytes

Capillary pericytes express an array of other ion channels, including the ubiquitous two-pore channels (TPCs), voltage-gated Na^+^ (Na_v_) channels, P2X receptors, and acid-sensing ion channels (ASICs; Table 1 and Figure 4). Due to their lower expression and dearth of functional data in capillary pericytes, detailed discussion of these channels is beyond the scope of this review, although we touch briefly upon the function of Piezo1 channels and P2X receptors.

### P2X Receptors

The ubiquitous purine ATP has received attention as a putative gliotransmitter (Pelligrino et al., 2011) and acts as an endogenous agonist at P2Y GPCRs and the cation-selective ionotropic P2X receptors, permeable to Na^+^, K^+^, and Ca^2+^ (Khakh et al., 2001). P2X receptors are trimmers consisting of intracellular N- and C-termini, a large extracellular domain containing the ATP binding site, and two transmembrane segments that line an integral ion pore (Kawate et al., 2009). Capillary pericytes express mRNA for P2X1 and P2X4 receptors (Table 1), which have a *p*Ca^2+^/*p*Na^+^ of ~5 and 4.2, respectively (Khakh et al., 2001). Thus, pericyte P2X receptors could function as sensors transducing ATP released into the local environment into Ca^2+^ elevations. Several studies have also suggested P2X7 receptors are functionally expressed in cultured human and freshly isolated rat retinal pericytes (Kawamura et al., 2003; Sugiyama et al., 2005; Platania et al., 2017), though it should be noted that our expression data do not unambiguously support the expression of this P2X isoform in CNS pericytes.

### Piezo1

Piezo1 is a large (2,521 amino acids in humans) mechanosensitive cation channel, with three identical subunits, thought to have 38 transmembrane segments, that form a central ion conduction pore with surrounding peripheral domains shaped like propeller blades (Coste et al., 2010; Zhao et al., 2016, 2018; Wu et al., 2017). Functional channels have a single channel conductance of 29 pS and a current that rapidly activates and then decays on a millisecond timescale (Coste et al., 2010, 2015; Zhao et al., 2018). In ECs, piezo1 can be activated by fluid shear stress, and has been implicated in blood flow regulation, vascular development and remodeling, and permeability (Li et al., 2014; Ranade et al., 2014; Friedrich et al., 2019). Piezo1 may play similar roles in capillary pericytes to mechanosensitive TRP channels in detecting changes in blood flow, vessel diameter, or paravascular fluid shear stress.

## A Birds-Eye View of Pericyte G-Protein Coupled Receptors

Pericytes express a huge variety of GPCRs (Table 2 and Figure 4) enabling them to transduce a vast array of extracellular stimuli into intracellular responses. As outlined above, many of the signaling pathways triggered by GPCR signaling impinge upon ion channel activity and thus regulate pericyte V_m_ and intracellular Ca^2+^.

Assessment of the general characteristics of the list of GPCRs expressed by pericytes is revealing. The majority of pericyte GPCRs primarily interact with G_i/o_ α subunits. This is closely followed by G_q_-coupled GPCRs, then those that are G_s_-coupled, and the remainder couple primarily to G_12/13_. Perhaps tellingly, expression of the *Gnas* gene, encoding the G_s_ α subunit, is ~5 times higher than those collectively encoding G_q/11_ α subunits, more than double that of G_i/o_ α subunit genes, and more than 12 times in excess of G_12/13_ genes (He et al., 2018; Vanlandewijck et al., 2018). Thus, while a wider variety of pericyte receptors may couple to depolarizing, Ca^2+^-elevating processes, it appears that hyperpolarizing G_s_ signaling may be a favored intracellular transduction pathway.

Around 12% of the receptor subtypes expressed by pericytes are promiscuous/pleiotropic in their G-protein coupling, the degree of which will depend on the expression levels of the signaling elements involved and their local densities and organization within GPCR signaling platforms. One such example is the highly-expressed A_2A_ adenosine receptor which couples primarily to G_s_, but also interacts with G_q_ and others (Olah, 1997; Fresco et al., 2004). Such promiscuity could represent an inbuilt feedback mechanism to prevent V_m_ being locked at hyperpolarized potentials by K^+^ channel activity, by facilitating recruitment of additional transduction pathways to promote repolarization. In contrast, the promiscuity in signaling exhibited among receptors that couple to G_q_, G_i/o_, and G_12/13_ would serve to reinforce depolarization. For example, the highly expressed S1P_3_ and PAR1 receptors frequently exhibit coupling to not just G_i/o_, but also to both G_q_ and G_12/13_ α subunits (Tobo et al., 2007; Means and Brown, 2009; Yue et al., 2012).

At the time of writing, a significant portion of GPCRs expressed by pericytes (Table 2) remain orphan receptors with little functional data available. Strikingly, one such orphan, GPRC5C, is the 4th most robustly expressed GPCR in these cells. Given this lack of data, we omit this group from further discussion.

## G-Protein Coupled Receptor Structure and Subclasses

The GPCR family represents the largest family of mammalian proteins (Lagerström and Schiöth, 2008; Katritch et al., 2014) sharing a common 7-transmembrane topology with an extracellular N-terminus and intracellular C-terminus. G-protein heterotrimers are organized into four principal categories based on the similarity of function and homology in their α subunits: G_s_, G_i/o_, G_q/11_, and G_12/13_ (Simon et al., 1991; Dupré et al., 2009). Broadly, the roles of these Gα subunits are to stimulate/inhibit production of cAMP by adenylate cyclase (AC; G_s_ and G_i/o_, respectively), to activate PLC (G_q/11_), and to activate Rho guanine nucleotide exchange factors (GEFs) (G_12/13_) (Hanlon and Andrew, 2015). The Gβγ subunit also activates downstream signaling elements and plays a role in GPCR mediated intracellular signaling (Dupré et al., 2009). Below, we outline how signaling through these pathways may modulate the activity of pericyte ion channel activity and consequently V_m_ and Ca^2+^ signaling, and we explore what the GPCRs expressed by pericytes might be able to tell us about NVC mechanisms.

## PKA as a G_s_- and G_i/o_-Controlled Modulator of Ion Channel Function

**Figure 9:**
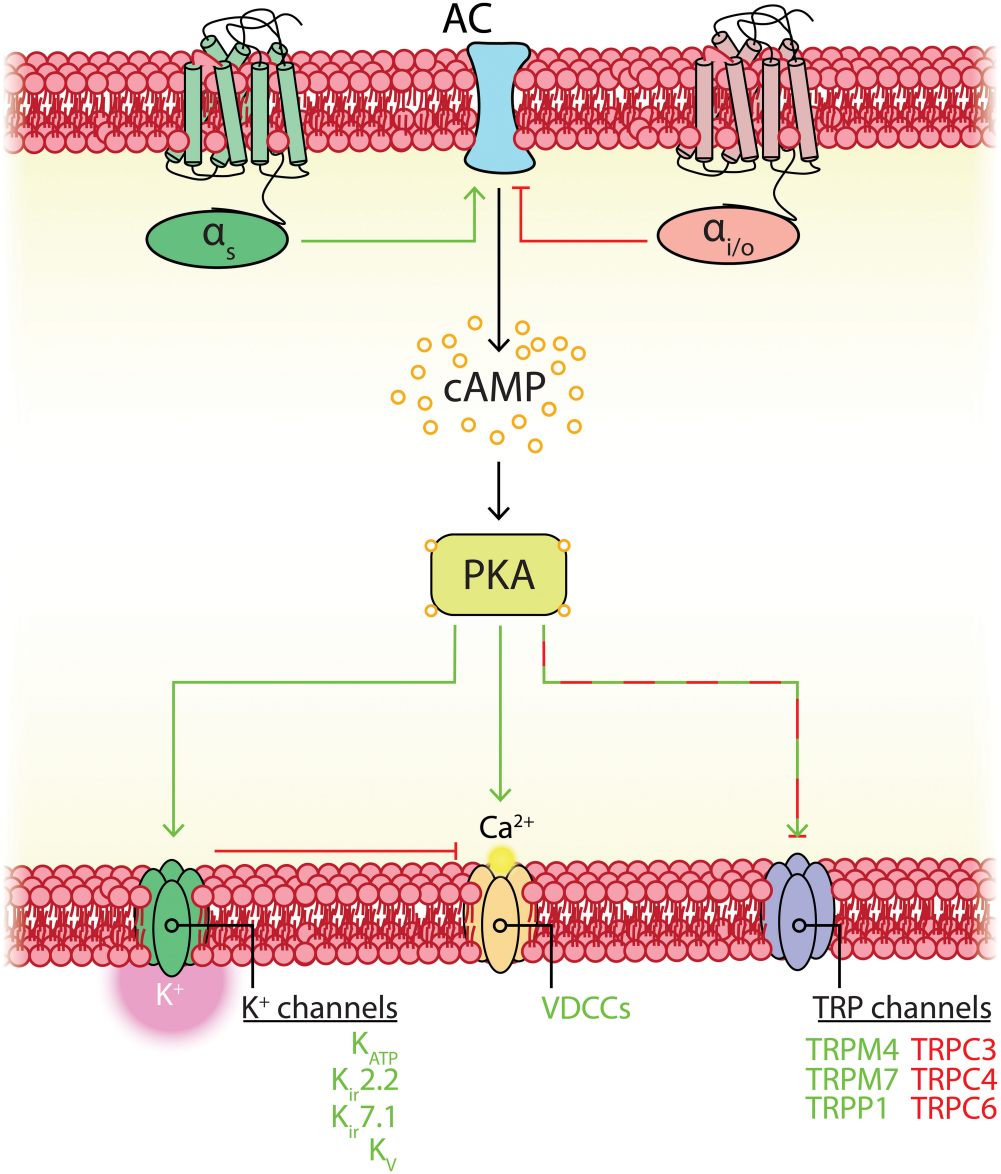
Potential G_s_- and G_i/o_-coupled GPCR–ion channel interactions in capillary pericytes. G_s_PCR activation promotes (green) adenylate cyclase (AC) activity, whereas G_i/o_PCR activation inhibits (red) AC. AC in turn generates cAMP from ATP, which stimulates PKA activity. PKA interacts with a broad range of ion channels. In pericytes, its activity is expected to couple to plasma membrane K^+^ and VDCC activity, with mixed effects on TRP channel activity. K^+^ channel hyperpolarization will oppose VDCC activity and thus the overall effect of G_s_ stimulation is membrane hyperpolarization.

In pericytes, G_s_ stimulation and subsequent PKA engagement is likely to drive phosphorylation of a number of ion channel targets including K_ATP_, a range of TRP channels, VDCCs, and IP_3_Rs—modulating their activity and thus V_m_ and cellular behavior (Figure 9). G_s_PCR activation leads to association of the Gα_s_ subunit with a cleft in the C2 domain of AC, catalyzing the conversion of ATP to cAMP (Sadana and Dessauer, 2009). cAMP then activates PKA by binding to its two regulatory subunits, inducing the dissociation of two catalytic subunits, enabling their subsequent phosphorylation of downstream targets (Sassone-Corsi, 2012). In contrast, G_i/o_ activation inhibits AC, opposing G_s_PCR activity. Here, Gα_i/o_ binds to the C1 domain of AC to inhibit enzymatic activity, although this is limited to the AC-I, -V, and -VI isoforms (Sadana and Dessauer, 2009).

### G_s_-cAMP-PKA Signaling Augments Hyperpolarizing K^+^ Currents in Pericytes

K_ir_ channels are likely key determinants of pericyte V_m_, and as noted previously K_ATP_ channel activity is bidirectionally modulated by cAMP levels. At tonic, low concentrations of cAMP, PKA increases vascular K_ATP_ channel activity by phosphorylating multiple sites on the pore-forming and regulatory subunits (Quinn et al., 2004; Shi et al., 2007, 2008b). At higher concentrations, cAMP conversely inhibits K_ATP_ channel activity in a Ca^2+^-dependent manner via engagement of the ubiquitous exchange protein activated by cAMP (Epac)-1 (Purves et al., 2009). PKA is preferentially activated by cAMP over Epac1, exhibiting a 30-fold lower EC_50_ (~1 vs. 30 μM; Purves et al., 2009). Accordingly, it seems that G_s_ activity will preferentially favor membrane hyperpolarization through K_ATP_ engagement. Consistent with this, activation of G_s_-coupled adenosine receptors leads to a dramatic increase in retinal pericyte K^+^ currents (Li and Puro, 2001). High-level accumulation of cAMP might in turn be expected to act as an inbuilt concentration-based feedback mechanism to inhibit the channel through Epac1 engagement.

In addition to such concentration-dependent regulation of channel activity, spatial considerations are important in determining the functional outcome of cAMP elevations. The assembly of ACs and phosphodiesterases into membrane-bound scaffolds organized around A-kinase anchoring proteins (AKAPs) has been suggested to facilitate the generation of microdomains of cAMP (Arora et al., 2013; Lefkimmiatis and Zaccolo, 2014). Such compartmentalization may facilitate specific, local adjustment of, for example, K_ATP_ channel activity in a select part of the cell (e.g., a thin-strand process or around a peg-socket junction in the case of pericytes) without impacting ion channels in other regions.

Complementary to the activation by PKA that K_ATP_ channels exhibit, K_ir_2.2 is also positively regulated by PKA (Zitron et al., 2004). Moreover, several K_v_ isoforms expressed by pericytes exhibit PKA sensitivity, in that the activity of K_v_7.4/7.5 heteromers or K_v_7.5 homomers is potentiated by PKA activation (Mani et al., 2016). K_v_2.1 membrane trafficking is also controlled by a PKA-dependent mechanism (Wu et al., 2015). Collectively, these data suggest a key stimulatory role for G_s_-cAMP-PKA signaling in the regulation of pericyte K^+^ channels, along with potential negative feedback mechanisms to prevent over-activation.

### G_s_-Mediated Reduction of TRP Channel Activity Complements K^+^ Channel Engagement

TRP channels are extensively regulated by G_s_ activity, and in contrast to K^+^ channels this typically leads to a decrease in activity. Focusing on the TRP isoforms expressed by pericytes, TRPC3, TRPC4, TRPC6, and TRPML1 are all inhibited by PKA phosphorylation (Vergarajauregui et al., 2008; Nishioka et al., 2011; Sung et al., 2011). In contrast, TRPM4 exhibits activation as a result of G_s_ stimulation in an Epac1-and IP_3_R-mediated Ca^2+^ release-dependent manner (Mironov and Skorova, 2011), and TRPM7 can also be potentiated by PKA (Takezawa et al., 2004). Phosphorylation of TRPP1 by PKA also increases channel P_o_ (Cantero del Rocío et al., 2015).

Thus, regulation of TRP channels via PKA is complex but it appears that this will to lean toward PKA-dependent inhibition of currents in pericytes. This reinforces the notion that engagement of PKA will shift the balance of ion channel activity to favor membrane hyperpolarization via K^+^ channel activity, while reducing Na^+^ and Ca^2+^ influx via TRP channels.

### G_s_ Activation May Promote Increases in Intracellular Ca^2+^

As noted, augmentation of Ca_v_1.2 is primarily dependent on PKA phosphorylation of Rad to relieve channel inhibition (Liu et al., 2020). PKA phosphoregulation of Ca_v_1.2 is also dependent on the AKAP isoform present in the macromolecular environment of the channel: AKAP15 permits sensitization of the channel whereas calcineurin associated with AKAP79 suppresses PKA-mediated increases in Ca_v_1.2 activity via dephosphorylation (Fuller et al., 2014). scRNAseq data (He et al., 2018; Vanlandewijck et al., 2018) indicate that pericytes express AKAP79 at low levels whilst expressing high levels of AKAP15, suggesting G_s_-stimulation in pericytes will favor increases in Ca_v_1.2 channel activity. Along similar lines, an increase in PKA activity induces sensitization of Ca_v_1.3 (Mahapatra et al., 2012), and Ca_v_3.1 currents are augmented in a cAMP/PKA-dependent manner (Li et al., 2012). Moreover, the current of Ca_v_3.2 is increased by cAMP, an effect that depends upon AKAP79/150, and its gene expression is also up-regulated by G_s_-signaling, suggesting a mechanism for long term T-type VDCC regulation (Liu et al., 2010; Sekiguchi and Kawabata, 2013). Accordingly, PKA activity should increase VDCC channel activity but, due to its voltage-dependence, in the broader context of the pericyte ion channel repertoire this must be weighed against simultaneous increases in activity of multiple K^+^ channels which will hyperpolarize V_m_ and keep VDCCs closed.

IP_3_Rs also possess phosphorylation sites for PKA (Ferris et al., 1991a; Vanderheyden et al., 2009) and can also be directly influenced by cAMP (Tovey et al., 2010), allowing for direct crosstalk between cAMP and Ca^2+^ release pathways. Indeed, phosphorylation by PKA induces an increase in sensitivity of the receptor for IP_3_, promoting IP_3_-induced Ca^2+^ release, while Epac1 activation also potentiates Ca^2+^ release (Vanderheyden et al., 2009; Mironov and Skorova, 2011).

Drawing all of these threads together, the complement of PKA targets and their relative expression levels in pericytes suggests that the G_s_-coupled receptors here likely primarily transduce stimuli into V_m_ hyperpolarization, but may in some cases also elevate intracellular Ca^2+^ via release from stores.

### The G_s_ Receptor Complement of Pericytes Suggest a Range of Potential Mediators for the Regulation of Blood Flow

Pericytes express a range of receptors that couple to G_s_–of particular note are the adenosine A_2A_ receptor, the PACAP receptor, PAC_1_, the prostacyclin IP receptor and the PTH-type 1 receptor (PTHR1). The expression of these suggests the possibility that their endogenous agonists could be released onto pericytes during neuronal activity to evoke membrane hyperpolarization and electrical signaling to increase blood flow (Figure 6).

The vasodilatory effects of adenosine, an abundant metabolic byproduct, have long been appreciated (Drury and Szent-Györgyi, 1929). In the brain, adenosine is released into the extracellular space by widely-expressed nucleoside transporters, or more commonly accumulates through the extracellular catabolism of ATP by ectonucleotidases (Cunha, 2016). Recent *in vivo* work showing a reliable correlation between extracellular adenosine accumulation and rapid increases in local O_2_ suggest that adenosine is capable of acting as a neurovascular coupling mediator (Wang and Venton, 2017), and clear links have been established between sensory stimulation, adenosine receptor engagement, and increases in cerebral blood flow (Ko et al., 1990; Dirnagl et al., 1994). The precise cellular and molecular mechanisms underlying this linkage remain to be determined, and actions through pericyte adenosine receptors are a strong candidate for mediating these effects.

Considering prostanoids also, blockade of G_s_-coupled IP receptors impairs neuronal activity-evoked vasodilation (Lacroix et al., 2015), which suggests a role for the classic vasodilator prostacyclin—produced in the same metabolic pathway as PGE_2_—in NVC. This possibility remains little explored, but the expression of IP receptors in pericytes provides a potential target for capillary endothelium-generated prostacyclin.

PACAP is a 27- or 38-amino acid neuropeptide that is an extremely potent vasodilatory agent (Koide et al., 2014). PACAP polypeptides are produced throughout the brain where they act as neurotransmitters and also have neurotrophic effects. These peptides are released by both neurons and astrocytes during activity and thus PACAP accumulation in the paravascular space could feasibly activate pericyte G_s_-coupled PAC_1_ receptors (Johnson et al., 2020), warranting further exploration of their potential involvement in NVC.

Finally, PTHR1 binds the endocrine ligand PTH and the paracrine ligand PTH-related protein-1 (PTHrP-1) (Vilardaga et al., 2011). Intriguingly, PTH binding to PTHR1 triggers sustained and prolonged cAMP production by retaining the intact ligand-receptor complex even after endocytosis (Ferrandon et al., 2009). This could have important implications for pericyte G_s_ signaling if PTH is released during neuronal activity.

### G_i/o_-Coupled P2Y_14_ Receptor Signaling May Impart Sensitivity to Local Metabolic Substrate Availability

The purinergic family P2Y_14_ receptor is the most robustly expressed GPCR in pericytes. This receptor signals through G_i/o_ and is activated by uridine diphosphate (UDP) and nucleotide sugars—most potently by UDP-glucose (Harden et al., 2010). UDP-glucose is synthesized from glucose and acts as a glucose donor in the synthesis of glycogen, which is present at modest levels in the brain (Leloir et al., 1959; Breckenridge and Crawford, 1960; Öz et al., 2015). This and related nucleotide sugars also act as donors for glycosylation in the ER lumen and Golgi apparatus (Berninsone and Hirschberg, 1998), and as a consequence these molecules are thought to be released under basal and simulated conditions from a broad range of cells, primarily through vesicular transport accompanying glycoconjugate delivery to the cell membrane (Harden et al., 2010; Lazarowski, 2012). The released nucleotide sugars have been hypothesized to act in an autocrine or paracrine manner on local P2Y_14_ receptors (Lazarowski and Harden, 2015), and as the hydrolyzation of UDP-glucose is three times slower than that of ATP, this has been suggested to result in long-duration signaling (Lazarowski, 2006). As its synthesis is dependent on glucose, we speculate that UDP-glucose signaling through P2Y_14_ may function to notify pericytes of local energy substrate availability: in conditions of ample glucose, UDP-glucose maintains activity of P2Y_14_, which through G_i/o_ signaling would counterbalance cAMP generation and prevent PKA activation of K_ATP_ and other K^+^ channels. In the event that glucose levels fall, such as during neuronal activity (Hu and Wilson, 1997; Paulson et al., 2010; Li and Freeman, 2015; Pearson-Leary and McNay, 2016) or in situations of metabolic stress, the loss of this negative feedback could be relieved, leading to cAMP elevations and engagement of K_ATP_ and other K^+^ channels to increase blood flow and replenish local glucose.

### mGluR_3_ and mGluR_7_ May Impart Glutamate Sensing Capabilities to Pericytes

The G_i/o_-coupled metabotropic glutamate receptors mGluR_3_ and mGluR_7_ are both localized in presynaptic terminals of GABAergic and glutamatergic synapses, and mGluR_3_ is also found in glia (Harrison et al., 2008; Palazzo et al., 2016). Like other mGluRs, these receptors contain a large N-terminal venus flytrap domain with a glutamate binding site that dimerizes with that of neighboring mGluRs. mGluR_7_ has a comparatively low affinity for glutamate and is thus activated only by its accumulation at high extracellular concentrations, but is also activated by elevations of intracellular Ca^2+^ through CaM interactions with its C-terminal tail. In neurons activity of these receptors exerts a hyperpolarizing influence that depresses synaptic activity through the lowering of cAMP, activation of G protein-coupled K_ir_ (GIRK) channels and the inhibition of VDCCs (Niswender and Conn, 2010). Pericytes do not express GIRKs, but they do express a range of VDCCs (Table 1). Thus, although the physiological roles of mGluRs in pericytes remain to be ascertained, their expression here implies that any glutamate elevations in the vicinity of pericytes could drive cAMP inhibition via mGluR_3_ and mGluR_7_ activation, and a reduction in Ca^2+^ entry via VDCCs.

## PKC Targets: G_q_-Dependent Modulation of Pericyte Ion Channels

**Figure 10:**
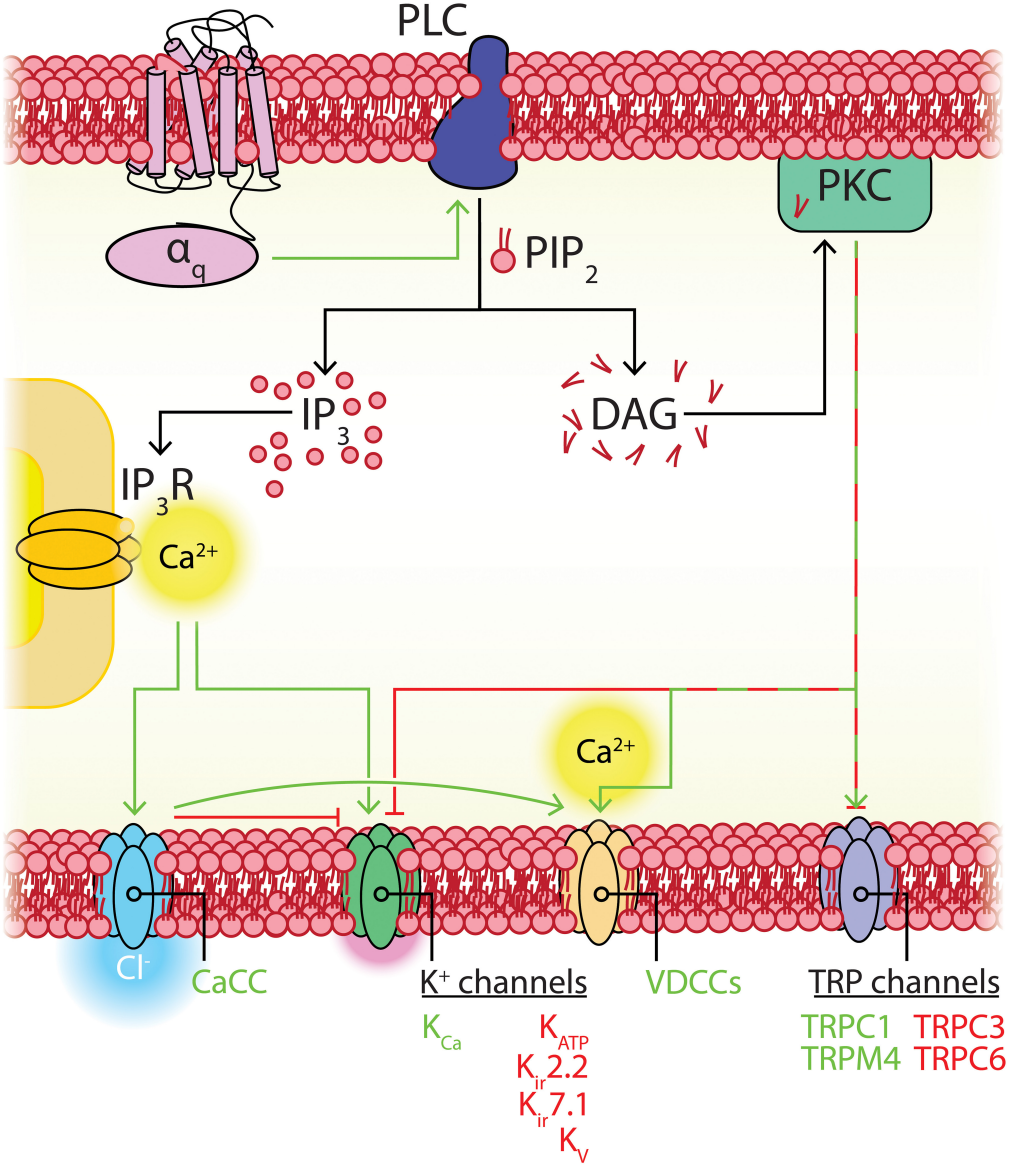
Potential G_q_PCR-ion channel interactions in capillary pericytes. G_q_PCR activation engages PLC, leading to the hydrolysis of PIP_2_ into IP_3_ and DAG. IP_3_ evokes Ca^2+^ release from the ER via resident IP_3_Rs, which may engage CaCCs and K_Ca_ channels. DAG stimulates PKC which has mixed effects on the TRP channels expressed by pericytes, promotes VDCC activity, and inhibits K_ATP_, K_ir_, and K_v_ channels. The net effect of engagement of G_q_PCRs is thus membrane depolarization and intracellular Ca^2+^ elevation.

Activation of the G_q_ α subunit stimulates phospholipase C (PLC), which mediates the conversion of membrane phospholipids to DAG and IP_3_, inducing PKC activation and Ca^2+^ release, respectively, which may affect a broad range of ion channels (Figure 10). We focus below on the ramifications of PKC signaling.

### G_q_-DAG-PKC Signaling Will Promote Depolarizing Currents in Pericytes

Activated PKC phosphorylates a diverse range of ion channels and is thus capable of exerting considerable influence on V_m_. PKCs are divided into three subfamilies depending on their activation requirements: conventional PKCs require DAG, Ca^2+^ and a phospholipid for activation; novel PKCs require DAG but are independent of Ca^2+^; atypical PKCs require neither of these (Newton, 2010). CNS capillary pericytes express PKC isoforms from each of these subfamilies (Table 3).

**Table 3:** Expression of PKC isoforms in brain capillary pericytes, and their modes of activation and regulation.

**PKC isoform**	**Gene name**	**Average counts/cell (annotated as a pericyte)**	**Class**	**Ca^**2+**^ activation**	**DAG activation**	**Phospholipid activation**	**Regulation by arachidonic acid**
PKC-α	*Prkca*	6.57	Conventional	Yes	Yes	Yes	+
PKC-β1	*Prkcb*	17.45	Conventional	Yes	Yes	Yes	+
PKC-β2	*Prkcb*		Conventional	Yes	Yes	Yes	+
PKC-γ	*Prkcg*	13.19	Conventional	Yes	Yes	Yes	+
PKC-δ	*Prkcd*	5.93	Novel	No	Yes	No	–
PKC-ε	*Prkce*	6.65	Novel	No	Yes	No	Insensitive
PKC-η	*Prkch*	1.25	Novel	No	Yes	No	+
PKC-θ	*Prkcq*	0.04	Novel	No	Yes	No	Insensitive
PKC-ι	*Prkci*	22.30	Atypical	Insensitive	Insensitive	Yes	Insensitive
PKC-ζ	*Prkcz*	0.06	Atypical	Insensitive	Insensitive	Yes	Insensitive

*mRNA average counts per cell were mined from He et al. (2018) and Vanlandewijck et al. (2018). For regulation by arachidonic acid, “+” denotes an increase in enzymatic activity, while “–” represents a decrease in enzymatic activity*.

All three IP_3_R isoforms can be phosphorylated by PKC. PKC phosphorylation of IP_3_R1 is potentiated by prior phosphorylation by PKA and increases Ca^2+^ release (Ferris et al., 1991a,b; Vermassen et al., 2004; Vanderheyden et al., 2009). In contrast, IP_3_R2 and IP_3_R3 are each inhibited by Ca^2+^-sensitive, conventional PKCs (Arguin et al., 2007; Caron et al., 2007).

K_ir_ channels are also extensively regulated by PKC, where phosphorylation inhibits K_ir_6.1-containing K_ATP_ channels, contrasting starkly with the stimulatory effects of PKA. This phosphorylation is graded—multiple serine residues (ser-354,−379,−385,−397, and−397 in the K_ir_6.1 C-terminal domain) can be phosphorylated, and the degree of inhibition is proportional to the number of these sites that receive a phosphoryl group from PKC (Shi et al., 2008b). In pericytes this graded response to PKC for the highly expressed K_ATP_ channel could provide a means to fine tune activity, by permitting the degree of local G_q_ signaling to oppose the stimulatory effects or PKA or ATP depletion. PKC also regulates the membrane density of K_ir_6.1, in that the PKCε isoform induces internalization of the receptor in a caveolin-dependent manner (Jiao et al., 2008), providing another avenue to decrease K_ATP_ channel activity. Likewise, K_ir_2.2 has multiple sites that inhibit channel current upon phosphorylation by PKC, but the graded PKC phosphorylation observed for K_ir_6.1 is absent (Kim et al., 2015; Scherer et al., 2016).

TRP channels are subject to complex regulation by G_q_ activity, with important roles for DAG, detailed above, and PKC. TRPC3 and TRPC6 in particular are inhibited by PKC despite activation by other elements of the G_q_ signaling cascade (Bousquet et al., 2010; Earley and Brayden, 2015), and TRPC1 is in contrast activated by PKC (Xiao et al., 2017). TRPM4 can be phosphorylated by PKC to sensitize the receptor to Ca^2+^ (Nilius et al., 2005), which augments Na^+^ entry in response to subsequent local Ca^2+^ elevations.

Ca_v_1.2 currents are enhanced by phosphorylation at Ser1928 by PKC isoforms from each subfamily (PKCα, PKCε, and PKCζ), permitting a broad range of conditions to regulate VDCC activity (Yang et al., 2005). As pericytes express members of all three subfamilies of PKC, regulation of Ca_v_1.2 activity may be similarly robust in these cells. Ca_v_1.2 surface expression is also increased within minutes of G_q_ stimulation via a PKC-dependent increase in channel trafficking to the plasma membrane (Raifman et al., 2017). In contrast, Ca_v_1.3 is negatively regulated by both conventional and atypical PKC isoforms (PKCβ2 and the PKCε, respectively), both of which are expressed in CNS pericytes (Table 3). As for T-type channels, Ca_v_3.1 activity is stimulated by PKC phosphorylation, independently of trafficking (Park et al., 2006), and Ca_v_3.2 is negatively regulated by Ca^2+^-independent PKCη phosphorylation (Zhang Y. et al., 2018), although PKCη is absent in pericytes.

PKCα also activates CaCCs to promote Cl^−^ efflux, where phosphorylation shifts the EC_50_ of intracellular Ca^2+^ from 349 to 63 nM for channel activation at −80 mV (Dutta et al., 2016).

Pulling these threads together, it seems that PKC activation as a result of G_q_ activity in pericytes will contrast with the effects of G_s_-cAMP-PKA signaling by enhancing activity of depolarizing ion channels such as VDCCs, TRP channels, and CaCCs while inhibiting hyperpolarizing channels such as K_ATP_ and K_ir_. Given that G_q_ activity also induces the release of Ca^2+^ from intracellular stores via IP_3_Rs, Ca^2+^-sensitive PKC activation may act as a further amplification loop to increase the signal:noise ratio of G_q_ signaling and promote Ca^2+^ accumulation and depolarization.

### Thromboxane and ET_A_ Receptors Are G_q_-Coupled Mediators of SMC Constriction That Are Robustly Expressed by Capillary Pericytes

The G_q_-coupled thromboxane (TP) receptor is well-known to induce vasoconstriction by SMCs (Dorn and Becker, 1993) and contractile mural cells of 1st−4th order vessels (Mishra et al., 2016). The TP receptor's endogenous agonists include a range of eicosanoid lipids that are generated from arachidonic acid (AA), which is initially mobilized from membrane phospholipid pools by the action of Ca^2+^-dependent phospholipase A_2_ (PLA_2_; Balsinde et al., 2002). Subsequently, cyclooxygenase or prostaglandin H_2_ (PGH_2_) synthase enzymes convert AA to PGH_2_, a potent agonist of the TP receptor. Further processing of PGH_2_ yields thromboxane-A_2_ (TxA_2_), a still more potent agonist (Bos et al., 2004; Woodward et al., 2011). Alternatively, AA can be shuttled down a cytochrome P450 ω-hydroxylase pathway to generate the TP agonist 20-HETE (Miyata and Roman, 2005). The contractile influence of 20-HETE has been suggested to play a major role in determining the diameter of cerebral arterioles and thus controlling brain blood flow (Attwell et al., 2010), and the activation of TP receptors has also been suggested to cause mild, slow contractions of capillary pericytes (Fernández-Klett et al., 2010). It is unknown whether pericyte TP receptors are basally active to produce this effect *in vivo*, but subtle changes in capillary diameter induced by this process could regulate local blood flow over the long term, dependent on the local levels of these agonists.

The ET_A_ receptor shares broad similarities with the TP receptor. Its principal transduction pathway is also G_q_—although coupling to other G proteins such as G_12/13_ has been noted—and similar to the TP receptor, its activation evokes robust SMC contractions (Sokolovsky, 1995; Horinouchi et al., 2013; Davenport et al., 2016). The agonist of the ET_A_ receptor, Endothelin-1, is constitutively released by ECs, SMCs, neurons and astrocytes (Russell and Davenport, 1999; Thorin and Webb, 2010; Freeman et al., 2014). In culture, release of endothelin-1 from ECs has been noted to drive changes in pericyte morphology through reorganization of F-actin and intermediate filaments (Dehouck et al., 1997), suggesting that ECs could regulate their coverage by pericyte processes through ET_A_ signaling. In the context of Alzheimer's disease, aberrant ET_A_ signaling caused by amyloid β accumulation results in capillary constriction by overlying pericytes which may limit oxygen and glucose delivery to the parenchyma (Nortley et al., 2019).

As described above, signaling through these receptors is expected to oppose G_s_-cAMP-PKA signaling while promoting membrane depolarization and elevation of Ca^2+^.

### Crosstalk and Control of G Protein Signaling Pathways

The preceding discussion illustrates that many channels expressed by pericytes are differentially regulated by PKA and PKC phosphorylation, and thus their activity will depend in part on the balance of activity between these pathways. Crosstalk between these pathways also occurs at the level of effectors, in addition to ultimate phosphorylation targets. For example, the G_q_ and G_i/o_ pathways oppose the G_s_ pathway at the level of AC, which can be Ca^2+^ sensitive and modulated by PKC, dependent on isoform (Chern, 2000). Indeed, the most highly expressed AC isoform in brain pericytes is ACVI (Table 4), which is regulated by PKC, G_i/o_, Ca^2+^, and Gβγ (Chern, 2000; Sadana and Dessauer, 2009). This regulation is mirrored for G_s_ acting on the G_q_ pathway, where PKA can directly inhibit the activity of PLC via phosphorylation (Nalli et al., 2014). Accordingly, G_s_- and G_q_-coupled receptors functionally oppose one another at multiple levels of their transduction pathways, which will help push the membrane potential toward either hyperpolarization or depolarization, respectively.

**Table 4:** Expression of isoforms of adenylate cyclase (AC) by CNS pericytes.

**AC isoform**	**Gene name**	**Average counts/cell**
		**(annotated as a pericyte)**
AC-I	*Adcy1*	0.79
AC-II	*Adcy2*	0.31
AC-III	*Adcy3*	15.18
AC-IV	*Adcy4*	11.33
AC-V	*Adcy5*	8.27
AC-VI	*Adcy6*	89.98
AC-VII	*Adcy7*	1.34
AC-VIII	*Adcy8*	0.05
AC-IX	*Adcy9*	55.32
AC-X	*Adcy10*	0.34

*mRNA average counts per cell were mined from He et al. (2018) and Vanlandewijck et al. (2018)*.

Another layer of control is provided by regulators of GPCR signaling (RGS)—small proteins that regulate the duration and intensity of GPCR signaling by driving GTPase activity of the Gα subunit and accelerating the hydrolysis of GTP, thereby inactivating their target (Ross and Wilkie, 2000; Kach et al., 2012). Capillary pericytes express high levels of RGS4 and 5 (Bondjers et al., 2003; He et al., 2018; Vanlandewijck et al., 2018) that act as GTPase activating proteins for G_i/o_ and G_q/11_ subunits, while seemingly sparing G_s_ (Berman et al., 1996; Watson et al., 1996; Hepler et al., 1997; Huang et al., 1997; Cho et al., 2003; Gunaje et al., 2011). Intriguingly, RGS4 is known to be phosphorylated by PKA and PKG, which stimulate its activity, accelerating the deactivation of G_q/11_ and inhibiting the hydrolysis of phosphoinositide to IP_3_ (Huang et al., 2007). Therefore, RGS engagement in pericytes may complement and amplify the hyperpolarizing effects of G_s_ signaling by stifling the depolarizing influences of G_i/o_ and G_q/11_.

## RhoA Targets: G_12/13_-Signaling

Capillary pericytes express several G_12/13_-coupled receptors, including a range of lysophospholipid receptors with important roles in lipid signaling, the promiscuous protease activated receptor PAR1, and several orphan receptors (Table 2). G_12/13_ activation couples to a number of interacting partners including cadherins, AKAPs, non-receptor tyrosine kinases and protein phosphatases, though its interaction with Ras homolog family member A (RhoA) is the best characterized (Worzfeld et al., 2008). In SMCs, RhoA engagement of its downstream effector Rho-associated kinase is known to contribute to a range of receptor-mediated contractile responses (Swärd et al., 2003).

RhoA is also frequently observed to be activated downstream of ion channel engagement, including TRPC6 and TRPM7 channels (Canales et al., 2019) and VDCCs (Fernández-Tenorio et al., 2011). RhoA modulating ion channel activity is less frequently reported, but RhoA may indirectly modulate V_m_ on slow time scales by promoting the endocytosis and translocation of channels such as K_v_1.2, IP_3_Rs, and TRPC1 (Mehta et al., 2003; Mayor and Pagano, 2007; Stirling et al., 2009) and possibly K_ATP_ channels (Foster and Coetzee, 2015). Effects of RhoA on K_ir_2.1 channel activity have also been reported, although the mechanistic details of this interaction have not been fully clarified (Jones, 2003).

## Gβγ Signaling and Pericyte Function

Initially, the Gβγ subunit was viewed as a negative regulator of the Gα subunit, serving to increase signal:noise ratio and specificity of signaling by preventing aberrant Gα activity in the absence of an agonist, but has since been found to be an active effector in its own right (Dupré et al., 2009), and may play important roles in pericyte physiology. Gβγ interacts with a range of canonical effectors (for example PLCβ, AC, GIRKs; Chern, 2000; Smrcka, 2008) along with a growing list of non-canonical effectors such as mitochondrial ATP synthase, a range of nuclear transcription factors, cytoskeletal regulators involved in motility, and constituents of the extracellular signal regulated kinase (ERK) pathway. These interactions implicate Gβγ in signaling roles as diverse as regulation of transcriptional activity, modulation of mRNA processing, control of nuclear import/export, cell motility, and oxidative phosphorylation (Khan et al., 2016). In addition to regulation of AC VI (Sadana and Dessauer, 2009)—the most highly expressed pericyte AC isoform (Table 4)—Gβγ signaling may also exert direct effects on pericyte V_m_ through activation of K_v_7.4 (Stott et al., 2015). In contrast Ca_v_2.1, Ca_v_3.2, and TRPM3 can be inhibited through Gβγ-dependent mechanisms (Hu et al., 2009; Zamponi and Currie, 2013; Alkhatib et al., 2019).

## Pericyte GPCRs that Couple to Multiple G proteins

The previously discussed GPCRs are largely selective in their G protein coupling, allowing for precise intracellular signaling in response to a range of stimuli. However, many GPCRs that are highly expressed in pericytes are capable of signaling through multiple G proteins. This may represent pleiotropy—physiological activation of different G proteins in response to differing signals—or promiscuity, i.e., engaging in non-preferred G protein interactions due to high levels of receptor expression or excessive stimulation (Maudsley et al., 2005). Here, we review examples of highly-expressed pericyte GPCRs with a tendency to couple to multiple G proteins.

### S1P Receptors

Sphingosine-1-phosphate (S1P) is a lipid mediator formed through the action of ceramidase on lipids of the plasma membrane (Ksiazek et al., 2015). S1P is constitutively released by erythrocytes and its plasma concentration strongly correlates with hematocrit (Selim et al., 2011; Ksiazek et al., 2015). The transporter-mediated release of S1P from ECs has also been documented (Kerage et al., 2014) along with the export of the enzyme that catalyzes its formation, sphingosine kinase (Ancellin et al., 2002). This leads to S1P signaling in the vasculature, which is particularly important for maintenance of the BBB (Janiurek et al., 2019), vasoconstriction (Salomone et al., 2010), angiogenesis, and regulation of vascular tone at the level of arterioles (Kerage et al., 2014).

Pericytes are ideally positioned to sense the release of S1P from local ECs. The actions of S1P are mediated through a family of receptors that act through G_i/o_, G_q_, and G_12/13_ signaling, with S1P_2_ and the robustly expressed S1P_3_ coupling to each of these (Means and Brown, 2009). Accordingly, S1P sensed by pericytes is expected to promote PLC engagement, Ca^2+^ elevations, a fall in cAMP, and depolarization, but further information as to the physiological roles of signaling through these receptors awaits experimental attention. As pericytes are critical for the maintenance of blood-brain barrier tightness (Armulik et al., 2010), it is possible that S1P signaling contributes to this process. S1P signaling also strengthens contact between ECs and pericytes in culture through a mechanism involving the trafficking and activation of the adhesion molecule N-cadherin by ECs (Paik et al., 2004), and it is thus possible that this is mirrored in pericytes to contribute to this interaction and maintain peg-socket junctions.

### PAR1 May Regulate Pericyte Thin-Strand Processes

Protease-activated receptor (PAR) 1 is a member of the PAR family and is stimulated by external proteases such as thrombin and trypsin. The proteolytic action of these enzymes on the extracellular domain of the receptor reveal an N-terminal tethered ligand sequence, exposure of which results in irreversible activity of the receptor that is halted only by its internalization (Soh et al., 2010). PARs are broadly expressed in the neurovascular unit, found in neurons, glia, ECs and SMCs, as well as pericytes. PAR1 couples to G_q_, G_i/o_, and G_12/13_, and while the release or activation of agonists for these receptors is typically associated with injury or inflammatory responses (Ma and Dorling, 2012; Yue et al., 2012), they have also been implicated in cell proliferation and differentiation, synaptic plasticity (Noorbakhsh et al., 2003), and driving vasodilation (Villari et al., 2017). Interestingly, thrombin signaling regulates morphology of fine processes in astrocytes through RhoA, and similar effects have been noted in neurons (Noorbakhsh et al., 2003). In line with this, it is possible that PAR1 signaling regulates the dynamics of pericyte process extension and retraction on capillaries (Berthiaume et al., 2018).

## Frizzled and Adhesion GPCRs in Pericytes

Finally, pericytes also express a range of members of the frizzled family of GPCRs. These are receptors for Wnt proteins, and G-protein coupling is of less importance in this group. Instead, canonical frizzled signaling occurs through the β-catenin pathway (MacDonald et al., 2009), but G protein coupling through signaling platforms assembled around the FZD-associated phosphoprotein Disheveled is also possible. The latter facilitates activation of G_q_- and G_i/o_-proteins to produce Ca^2+^ elevations and PKC engagement (Schulte, 2010; Kilander et al., 2014). Further research is required to infer the functional implications of pericyte expression of frizzled receptors, but developmental and homeostatic roles seem likely, as these are major aspects of Wnt signaling (Yang, 2012). Low levels of the adhesion class cadherin EGF lag seven pass receptors (CELSR)2 are also seen in pericytes.

## Control of Pericyte V_m_ by Pericyte Ion Channels and GPCRs—Conclusions and Future Perspectives

The ion channels and GPCRs expressed by capillary pericytes represent a toolkit for the dynamic control of pericyte membrane potential and function. Among a panoply of roles for these signaling elements, the robust expression of genes encoding K^+^ channels and G_s_PCRs and their second messenger components implies an important role for pericyte membrane hyperpolarization, which we suggest contributes to long-range electrical signaling to control blood flow (Figure 6). Importantly, disturbances in blood flow and the processes that regulate it are increasingly appreciated to play a key role in a variety of pathological conditions. These include dementias such as Alzheimer's disease (AD) (Alsop et al., 2000; Iadecola, 2004; Nicolakakis and Hamel, 2011; Iturria-Medina et al., 2016), small vessel disease of the brain (Dabertrand et al., 2015; Capone et al., 2016; Huneau et al., 2018), psychological conditions such as schizophrenia (Mathew et al., 1988; Zhu et al., 2017) and chronic stress (Longden et al., 2014; Han et al., 2019), plus diabetes (Mogi and Horiuchi, 2011; Vetri et al., 2012), hypertension (Girouard and Iadecola, 2006; Capone et al., 2012), and stroke (Girouard and Iadecola, 2006; Koide et al., 2012; Balbi et al., 2017), and pericytes appear to be exceptionally sensitive to pathological perturbations (Winkler et al., 2011).

The ion channels and GPCRs that are highly expressed by brain pericytes thus have the potential to be pharmacological targets for vascular disorders, metabolic diseases, and neurodegenerative and neurological disorders (wherein for example K_ATP_ channels, IP_3_Rs, VDCCs, TRP channels, and GPCRs such as A_2A_ and ET_A_ receptors have been implicated, to name but a few; Hübner and Jentsch, 2002; Jacobson and Gao, 2006; Nilius et al., 2007; Ohkita et al., 2012; Aziz et al., 2014; Mikoshiba, 2015). Thus, furthering our understanding of the mechanisms through which pericytes contribute to blood flow control in the brain is a critical step in the search for ways in which to prevent decline or restore function in these disease contexts. The data we have discussed underscore that we are at an early stage in our understanding of how pericyte ion channels and GPCRs contribute to these functions, and warrant further studies to reveal novel mechanisms and therapeutic targets.

In the future, it will be important to determine the precise effects of both hyperpolarization and depolarization on pericyte functional outputs, for which optogenetic technologies or traditional electrophysiological approaches (Zhang et al., 2011) can be leveraged. At a deeper level, questions regarding the organization of pericyte ion channels and GPCRs await exploration—are these organized into macromolecular signaling complexes to facilitate privileged communication between complementary molecular players? Are these elements concentrated at sites to optimize cell-cell communication, such as peg-socket junctions, or distributed more broadly throughout the cell? What are the mechanisms that modulate the fidelity and gain of signaling (control of gene expression, protein trafficking, cell surface expression levels, and so on) and how are these affected in cerebrovascular disorders? The present survey of pericyte ion channels and GPCRs provides a map that can be used to guide these deeper explorations.

## Author Contributions

All authors listed have made a substantial, direct and intellectual contribution to the work, and approved it for publication.

## Conflict of Interest

The authors declare that the research was conducted in the absence of any commercial or financial relationships that could be construed as a potential conflict of interest.
